# Differential Abundance Analysis with Bayes Shrinkage Estimation of Variance (DASEV) for Zero-Inflated Proteomic and Metabolomic Data

**DOI:** 10.1038/s41598-020-57470-4

**Published:** 2020-01-21

**Authors:** Zhengyan Huang, Andrew N. Lane, Teresa W-M. Fan, Richard M. Higashi, Heidi L. Weiss, Xiangrong Yin, Chi Wang

**Affiliations:** 1 Department of Biostatistics, University of Kentucky, Lexington, Kentucky 40536 USA; 2 Markey Cancer Center, University of Kentucky, Lexington, Kentucky 40536 USA; 3 Center for Environmental and Systems Biochemistry, University of Kentucky, Lexington, Kentucky 40536 USA; 4 Department of Toxicology and Cancer Biology, University of Kentucky, Lexington, Kentucky 40536 USA; 5 Department of Statistics, University of Kentucky, Lexington, Kentucky 40536 USA

**Keywords:** Metabolomics, Metabolomics, Proteomics, Proteomics, Statistics

## Abstract

Mass spectrometry (MS) is frequently used for proteomic and metabolomic profiling of biological samples. Data obtained by MS are often zero-inflated. Those zero values are called point mass values (PMVs). Zero values can be further grouped into biological PMVs and technical PMVs. The former type is caused by true absence of a compound and the later type is caused by a technical detection limit. Methods based on a mixture model have been developed to separate the two types of zeros and to perform differential abundance analysis comparing proteomic/metabolomic profiles between different groups of subjects. However, we notice that those methods may give unstable estimate of the model variance, and thus lead to false positive and false negative results when the number of non-zero values is small. In this paper, we propose a new differential abundance analysis method, DASEV, which uses an empirical Bayes shrinkage method to more robustly estimate the variance and enhance the accuracy of differential abundance analysis. Simulation studies and real data analysis show that DASEV substantially improves parameter estimation of the mixture model and outperforms current methods in identifying differentially abundant features.

## Introduction

In recent years, many proteomic and metabolomic studies have been performed to understand diseases’ biological mechanisms, to identify prognostic and predictive biomarkers, and to develop better treatments^1,2^. A widely used platform for proteomic and metabolomic profiling is mass spectrometry (MS). The high-throughput data produced by MS are often zero-inflated. These zero values are called point mass values (PMVs)^3^. Depending on the origin of the zeros, PMVs can be further classified into biological PMVs (BPMVs), where the compound is absent from the sample, and technical PMVs (TPMVs), where the compound is present but its abundance is below the detection limit of the particular instrument used^3,4^. The proportion of PMVs can be very large^3,5^. Two examples in case are briefed here with more detailed descriptions in the Results section. The first example is a human urinary proteomic dataset of over 1,800 subjects with 5,270 features^6^. The average PMV proportion among all features is 81%. There are 2,537 features having PMVs in more than 90% of subjects. The second example is an exosomal lipids dataset of 91 lung cancer subjects with 101 features^7^. It has an average PMV proportion of 82%. There are 56 features having PMVs in more than 90% of subjects. Due to the large amount of PMVs, it is critical for downstream statistical analysis to account for them, preferably further distinguishing BPMVs and TPMVs, to ensure unbiased and efficient inference.

Identifying differentially abundant features between experimental groups or disease phenotypes is central for many proteomic and metabolomic studies. Several statistical methods have been proposed for differential abundance analysis based on zero-inflated MS data. Gleiss *et al*.^3^ classified these methods into three types. The first type of methods is one-part tests, which consider the data as left-censored and use a single model to characterize it. For example, an adaptive t-test imputes PMVs with certain non-zero values and performs a two-sample t-test on imputed data^3^. As another example, a Tobit model assumes the data follows a left-censored normal distribution and use a likelihood ratio test to compare distributional parameters between groups^3,8^. The second type of methods is two-part tests, which models PMVs and non-PMVs separately. For example, a two-part t-test employs a first statistic to compare the proportion of PMVs and a second statistic to compare the non-PMV values between groups. These two test statistics are then combined to calculate the p-value. One limitation of these two types of methods is that they do not distinguish TPMVs and BPMVs. To address this problem, a third type of methods has been proposed by Taylor *et al*.^5^ and Gleiss *et al*.^3^ based on a mixture model. The mixture model explicitly characterizes PMVs as a mixture of TPMVs and BPMVs. The BPMVs are considered as a point mass at zero. The non-BPMVs, including both TPMVs and non-PMVs, are considered as coming from a lognormal distribution, where the data are left-censored at the detection limit. A likelihood ratio test is proposed to test whether both the proportion of point mass at zero and the mean parameter of the normal distribution are the same between experimental groups. Taylor *et al*.^5^ and Gleiss *et al*.^3^ compared the performance of the mixture model to many one-part and two-parts tests and concluded that the mixture model was preferred in parameter estimation in presence of TPMVs, especially when the proportion of TPMVs was large.

**Figure 1:**
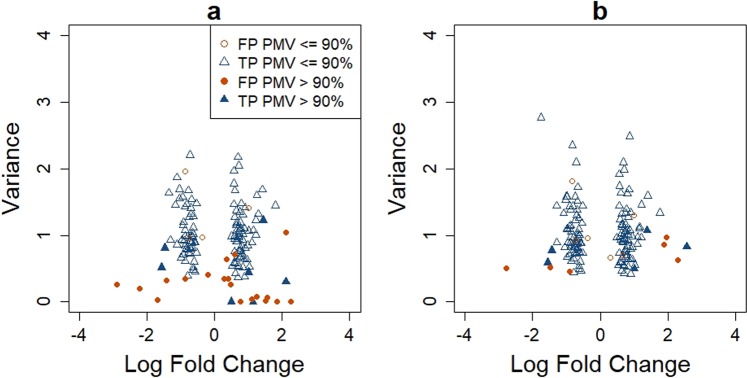
Estimated log fold change versus variance based on TLK (panel a) and DASEV (panel b) for 150 top-ranked features from a two-group differential abundance analysis. Data were simulated from the first simulation scenario as described in the Simulation Studies section with a sample size of 200 per group. Features were ranked based on their p-values. FP: false positive; TP: true positive.

Although the mixture model is appealing in distinguishing TPMVs and BPMVs and therefore providing better characterization of MS data, parameter estimations, especially the variance paramter estimation, from the model are unstable in presence of large proportion of zero values. The instability in variance parameter estimation substantially affects differential abundance analysis results. On the one hand, overestimation of the variance may lead to false negative results. On the other hand, underestimation of the variance may lead to false positive results. To demonstrate the impact of underestimating the variance on differential abundance analysis, Fig. 1a shows the top-ranked 150 features based on the mixture model in Taylor *et al*.^5^ (referred to as the TLK method) from a simulated two-group comparison dataset. The data simulation procedure is provided in the Simulation Studies section. There were more than 20 false positive features. Most of these features had large fractions of zero values (>90%) and very small variance estimates from TLK. As a result, these features got favorable rankings even though some of them had very small fold changes.

We hereby propose a new method called Differential Abundance analysis with Shrinkage Estimation of Variance (DASEV). Our method is based on the mixture model but provides a more robust estimation of model parameters. Specifically, an empirical Bayes shrinkage method is proposed for borrowing information across the ensemble of proteomic or metabolomic features to better estimate the variance parameter of each feature. Bayes and empirical Bayes shrinkage methods have been shown to provide robust estimation of model parameters, especially the variance parameter, for microarray^9^, RNAseq^10,11^, ChIPseq^12^, and NanoString nCounter^13^ data. To our knowledge, this paper is the first to introduce such method to the mixture model for MS-based proteomic or metabolomic studies. Based on the mixture model and stabilized variance estimate, we propose three statistical tests to examine whether the BPMV proportion, the mean of non-BPMVs, or both are the same between patient groups. Simulation studies and real data analysis demonstrate that our method substantially improves parameter estimation of the mixture model and outperforms current methods in identifying differentially abundant proteomic/metabolomic features.

## Methods

### The model

The MS data for a proteomic or metabolomic feature contains non-PMVs and PMVs, where PMVs are a mixture of BPMVs and TPMVs. We assume that non-BPMVs follow a lognormal distribution, i.e. the logarithm of non-BPMVs follow a normal distribution. Due to the detection limit, non-BPMVs values below the detection limit are censored, leading to TPMVs. Therefore, the observed abundance of feature *k* for subject *i*, *Y*_*ik*_, follows a mixture distribution^3,5^ with the following density function

1 $$f({Y}_{ik}|{\gamma }_{k},{\beta }_{k},{\sigma }_{k};{\lambda }_{k})=\{\begin{array}{cc}{p}_{ik}+(1-{p}_{ik})\Phi \{({\lambda }_{k}-{\mu }_{ik})/{\sigma }_{k}^{2}\}, & if\,PMVs\\ (1-{p}_{ik})\phi [\{log({Y}_{ik})-{\mu }_{ik}\}/{\sigma }_{k}^{2}], & if\,non-PMVs\end{array}$$

where *p*_*ik*_ is the proportion of BPMVs and *μ*_*ik*_ is the mean of non-BPMVs for feature *k* in subject *i*, *σ*_*k*_ is the standard deviation, *λ*_*k*_ is the logarithm of the detection limit for feature k, and Φ and *ϕ* are cumulative distribution and density functions of a standard normal distribution, respectively. The logarithm of detection limit, *λ*_*k*_, is usually set as the minimal log-transformed non-PMV observations minus a small number *ϵ*. In this paper, we set *ϵ* equal to 0.1, which was also used in Gleiss *et al*.^3^. To quantify associations between the feature abundance and a vector of covariates *X*_*i*_, we further assume a logistic regression model for *p*_*ik*_ and a linear regression model for *μ*_*ik*_ as follows

$$log\left(\frac{{p}_{ik}}{1-{p}_{ik}}\right)={X}_{i}^{T}{\gamma }_{k},\ and\ {\mu }_{ik}={X}_{i}^{T}{\beta }_{k},$$

where *γ*_*k*_ and *β*_*k*_ are vectors of regression coefficients quantifying the covariates’ effects on the mean of logarithm of non-BPMVs and the proportion of BPMVs, respectively. Note that we use the same vector of covariates in the two models. But the method can be easily generalized to allow different vectors of covariates for *p*_*ik*_ and *μ*_*ik*_.

**Figure 2:**
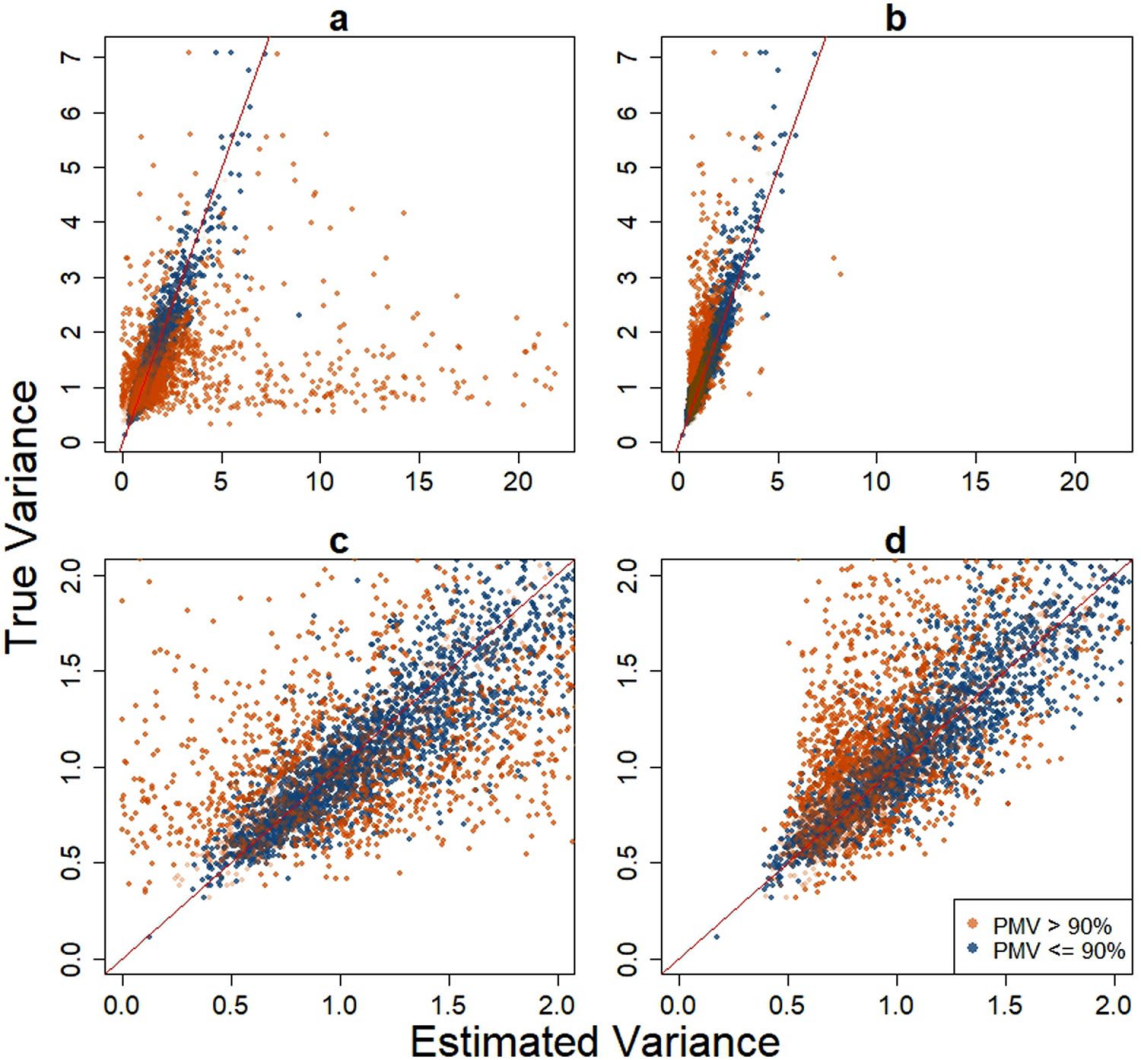
Comparison of estimated variance versus true variance for TLK (panels a and c) and DASEV (panels b and d) based on a single simulation with sample size 200 per group from the first scenario. Panels c and d are magnified lower left corner of panels a and b, respectively. The red line shows where the estimated variance equals the true variance.

**Figure 3:**
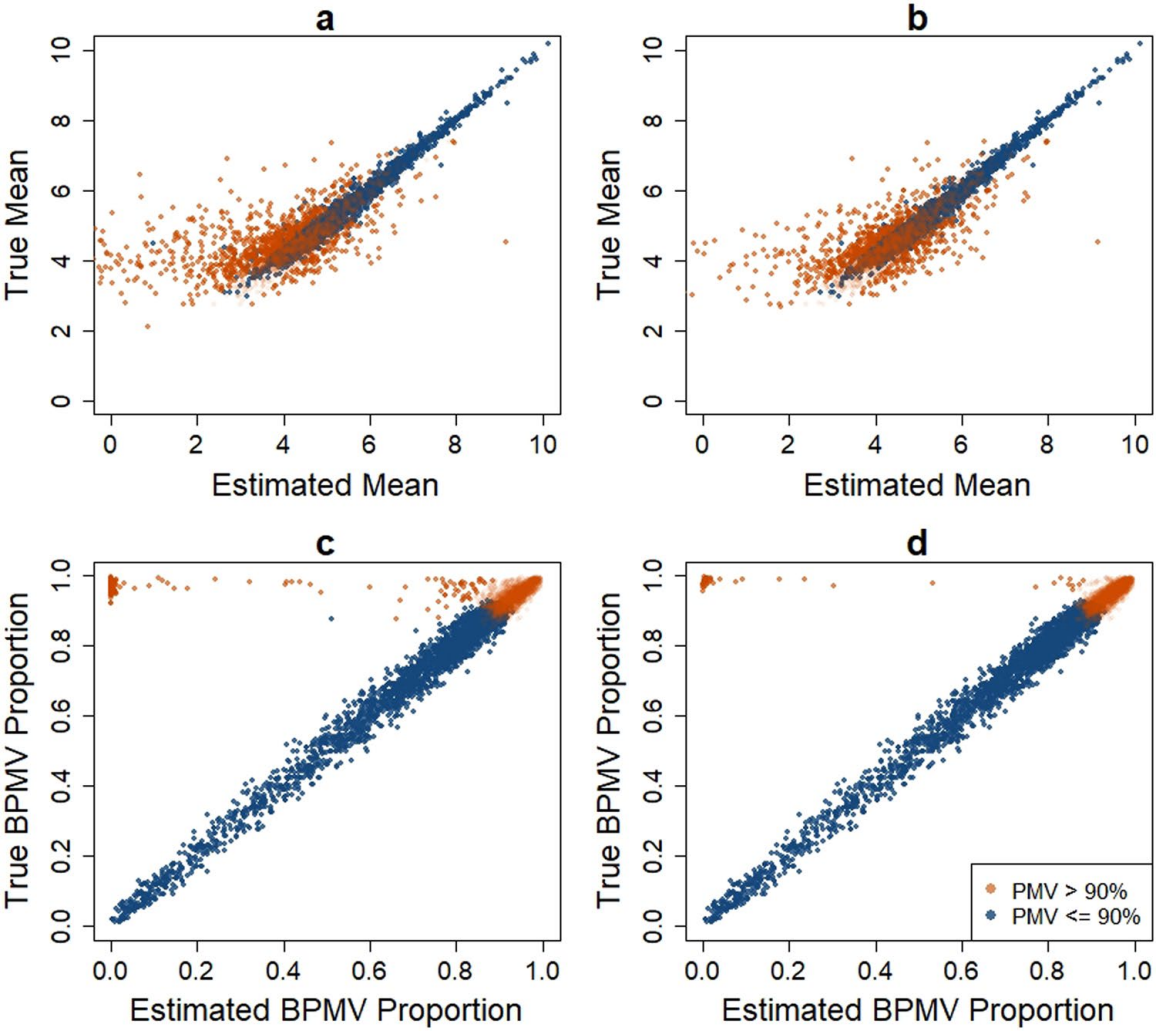
Comparison of estimated non-BPMV mean and BPMV proportion versus true values for TLK (panels a and c) and DASEV (panels b and d) based on a single simulation with a sample size of 200 per group from the first scenario. This figure only shows results for control group. Case group has identical patterns.

In presence of a large fraction of zero values, the parameter estimation based on model (1) can be unstable (Figs. 2 and 3). We consider an empirical Bayes shrinkage method to improve the estimation. The empirical Bayes shrinkage method has been shown to provide robust estimation of the variance parameter in the analysis of other types of omics data^9–13^. To our knowledge, this paper is the first to introduce such method to the mixture model for MS-based proteomic or metabolomic studies. Specifically, we consider the following prior distribution for ${\sigma }_{k}^{2}$ across all features:

$${\sigma }_{k}^{2} \sim Inv-Gamma({d}_{0}/2,\,{d}_{0}{s}_{0}^{2}/2),$$

where *d*_0_/2 and ${d}_{0}{s}_{0}^{2}$/2 are the shape and scale parameters of the inverse-gamma distribution, respectively. As shown in Supplementary Fig. S1, this prior fits the empirical distribution of estimated variances from the human urinary proteome dataset. This prior distribution allows us to borrow information across the ensemble of features so that more stable variance estimation can be obtained. The hyperparameters of the prior distribution, *d*_0_ and *s*_0_, are determined as described in the next subsection.

### Determining hyperparameters

The hyperparameters are empirically determined from a rough estimate of feature variance based on the observed data. From Eq. (1), the non-PMV observations follow a truncated normal distribution $f({Y}_{ik}| {Y}_{ik} > 0,{\beta }_{k},{\sigma }_{k};{\lambda }_{k})=\phi \{log({Y}_{ik})-{\mu }_{ik}/{\sigma }_{k}^{2}\}/{\sigma }_{k}/[1-\Phi \{({\lambda }_{k}-{\mu }_{ik})/{\sigma }_{k}^{2}\}]$. A rough estimate of *σ*_*k*_, ${\widehat{\sigma }}_{k}$, is obtained by maximizing the likelihood for non-PMVs ${\Pi }_{i=1}^{n}f({Y}_{ik}| {Y}_{ik}\  > \ 0,{\beta }_{k},{\sigma }_{k};{\lambda }_{k})$, where *β*_*k*_ is set as the parameter vector for sample mean of non-PMVs, *n* is the number of observations. Note that ${\widehat{\sigma }}_{k}$ is only used for the calculation of hyperparameters. Once the hyperparameters are determined, a more robust estimate of *σ*_*k*_ is obtained and used for model inference (see next subsections). Let *m* and *v* be the sample mean and variance for ${\widehat{\sigma }}_{k}$ across features, respectively. Based on the inverse gamma distribution and using the method of moments (see Supplementary Materials for the derivation), the *d*_0_ and *s*_0_ are calculated as follows

$${d}_{0}=\frac{2{m}^{2}}{v}+4,\ and\ {s}_{0}=\sqrt{\frac{m({d}_{0}-2)}{{d}_{0}}}.$$

In order to robustly estimate *d*_0_ and *s*_0_, we restrict features included in this calculation to be those with at least 10 non-PMV observations from the two groups combined. But if the number of such features is less than 30, we use the top 30 features with the smallest PMV proportions.

### Model parameter estimation

We use an iterative procedure to obtain our estimates of ${\theta }_{k}={({\gamma }_{k}^{T},{\beta }_{k}^{T})}^{T}$ and *σ*_*k*_. For a given *σ*_*k*_, *θ*_*k*_ is estimated by maximizing the likelihood

2 $$L({\theta }_{k}| {\sigma }_{k})=\mathop{\prod }\limits_{i=1}^{n}f({Y}_{ik}| {\gamma }_{k},{\beta }_{k},{\sigma }_{k};{\lambda }_{k}).$$

For a given *θ*_*k*_, *σ*_*k*_ is estimated by maximizing the posterior

3 $$p({\sigma }_{k}^{2}| data)\propto \mathop{\prod }\limits_{i=1}^{n}f({Y}_{ik}| {\gamma }_{k},{\beta }_{k},{\sigma }_{k};{\lambda }_{k})\times \pi ({\sigma }_{k}^{2}| {d}_{0},{s}_{0}),$$

where $\pi ({\sigma }_{k}^{2}| {d}_{0},{s}_{0})={({d}_{0}{s}_{0}^{2}/2)}^{{d}_{0}/2}{\sigma }_{k}^{-2(1+{d}_{0}/2)}\exp \{-{d}_{0}{s}_{0}^{2}/(2{\sigma }_{k}^{2})\}/\Gamma ({d}_{0}/2)$ is the prior inverse-gamma density of ${\sigma }_{k}^{2}$. Note that Eq. (3) can also be viewed as a penalized likelihood with the term $\pi ({\sigma }_{k}^{2}| {d}_{0},{s}_{0})$ to penalize values far from the prior mean of ${\sigma }_{k}^{2}$.

We start with ${\widehat{\sigma }}_{k}$ as the initial value of *σ*_*k*_, plug it into Eq. (2) to find the maximum likelihood estimate of *θ*_*k*_, ${\widehat{\theta }}_{k}$. The ${\widehat{\theta }}_{k}$ is then plugged into Eq. (3) to obtain the posterior mode, ${\widetilde{\sigma }}_{k}$. By iteratively updating ${\widehat{\theta }}_{k}$ and ${\widetilde{\sigma }}_{k}$ until convergence, we obtain estimates of *θ*_*k*_ and *σ*_*k*_.

### Hypothesis testing

For each feature, our method allows examining the following three hypotheses regarding the effect of the *j*^*th*^ covariate:

$${H}_{0}^{M}:{\beta }_{jk}=0\ vs\ {H}_{A}^{M}:{\beta }_{jk}\ne 0,$$

which tests if the covariate has an effect on mean abundance.

$${H}_{0}^{P}:{\gamma }_{jk}=0\ vs\ {H}_{A}^{P}:{\gamma }_{jk}\ne 0,\,$$

which tests if the covariate has an effect on BPMV proportion.

$${H}_{0}^{B}:{\beta }_{jk}=0\ and\ {\gamma }_{jk}=0\ vs\ {H}_{A}^{B}:{\beta }_{jk}\ne 0\ or\ {\gamma }_{jk}\ne 0,$$

which tests if the covariate has an effect on mean abundance or BPMV proportion. A likelihood ratio test statistic is used to test each of the three hypotheses. The test is based on the ratio of the maximum likelihood under the null hypothesis and that without the constraint, where the maximum likelihood is calculated based on the procedure described in the previous subsection with *σ*_*k*_ estimated by ${\widetilde{\sigma }}_{k}$. A p-value is obtained based on a *χ*^2^ distribution with one (for testing ${H}_{0}^{M}$ or ${H}_{0}^{P}$) or two (for testing ${H}_{0}^{B}$) degrees of freedom. The Benjamini and Hochberg procedure^14^ is used to control the false discovery rate (FDR) across features.

## Results

To get a comprehensive evaluation of the performance of DASEV, and to compare with TLK, we used a combination of simulations and real data. For TLK, the algorithm returns a p-value for each feature, but does not provide a procedure for multiple comparisons adjustment. To allow a fair comparison, we used the Benjamini-Hochberg procedure^14^ to control the FDR, the same as we used in DASEV.

### Simulation studies

#### Simulation settings

To mimic real-world situation, we based our simulation on real data obtained from the human urinary proteome database. The dataset we used contains two groups (benign prostatic hyperplasia patients and healthy controls). A detailed description of the dataset is provided in the human urinary proteome data analysis subsection. We applied DASEV to this dataset and estimated feature-wise mean of non-BPMVs in the control group, BPMV proportion in the control group, detection limit, and variance. We filtered out features with the estimated group mean smaller than the detection limit. The estimated BPMV proportions range from 0.6% to 99.9% with 80.6% as the mean value. The estimated percentages of TPMVs range from 0% to 47.2% with 1.6% as the mean value. There were 971 features having q-values less than 0.01, in which 275 (28.3%) features had estimated fold changes ≥ 2. For our simulations, each simulated dataset contained 5,000 features for two groups (case and control). The abundance level of a feature was simulated based on the mixture distribution in Eq. (1), where the model parameter values were randomly resampled from the parameter estimates of the control group of the real data. Since mean, BPMV proportion and detection limit were correlated, those values were resampled together. The variance parameter was resampled separately because we did not observe a correlation between variance and other parameters. We considered six differential abundance scenarios to evaluate the method’s performance, see Supplementary Table S1 for detailed settings for all simulation scenarios. Part of results from the first scenario are presented in the next subsection, while all other results were presented in Supplementary Materials. Our first scenario aimed to evaluate methods’ performance in identifying the non-BPMV mean difference between groups. We randomly selected 10% of features to have a 2-fold change in the mean abundance of non-BPMVs between case and control groups, with 5% of the features having higher abundance and another 5% having lower abundance in the case group. We considered sample sizes of 10, 20, 100 or 200 per group and replicated the simulation 100 times for each sample size. For each simulated dataset, we focused our analysis on features with at least three non-PMV observations and at least one PMV and one non-PMV observation per group. Results from sample size of 100 and 200 are presented in the following subsection. Results from sample size of 10 and 20 are presented in Supplementary Materials.

#### Simulation results

We first examined whether our simulated data represent distributions of BPMV and TPMV proportions in the real data. Among 100 simulation replicates with a sample size of 200 per group, the BPMV proportion for the control group ranged from 0.7% to 99.9% with 79.6% as the mean value. The percentage of TPMVs for the control group ranged from 0% to 48.1% with 1.5% as the mean value. Those percentages were close to the values estimated from the human urinary proteome data. For differentially abundant features, the added fold changes affected TPMV proportions in the case group. Among features with higher abundance in the case group, the TPMV percentage ranged from 0% to 29.5%. Among features with lower abundance in the case group, the TPMV percentage ranged from 0% to 74.9%.

Under the first simulation scenario, we first compared the parameter estimation between DASEV and TLK. Figure 2 presents the feature variance estimation from DASEV and TLK based on a simulated dataset. The TLK variance estimation was unstable. On one side, TLK substantially overestimated the variance for a subset of features. As shown in Fig. 2a, over 100 features had estimated variance 10 times larger than the true value. On the other side, TLK substantially underestimated the variance for another subset of features. As shown in Fig. 2c, 83 features had estimated variance smaller than 0.3. Majority of those extreme estimations were for features with large fractions of PMVs. In contrast, DASEV provided a much more robust estimation of the feature variance. It seldom gave extremely large (Fig. 2b) or small (Fig. 2d) variance estimates.

The unstable estimate of variance based on TLK also affected the estimation of mean and BPMV proportion. As shown in Fig. 3a,c, the estimated mean and BPMV proportion were far from true values for a subset of features, especially features with large fractions of PMVs. Among features with over 90% PMVs, 13.4% had the estimated mean less than the detection limit. In contrast, DASEV yielded much fewer features (2.4%) with deviated mean and BPMV proportion estimates (Fig. 3b,d). The figure only shows results for control group. Case group has identical patterns. We obtained similar results on parameter estimation from simulations with 100 observations per group (Supplementary Figs. S2, S3, and S4). Parameter estimation from other simulation scenarios showed similar patterns (results not shown).

**Figure 4:**
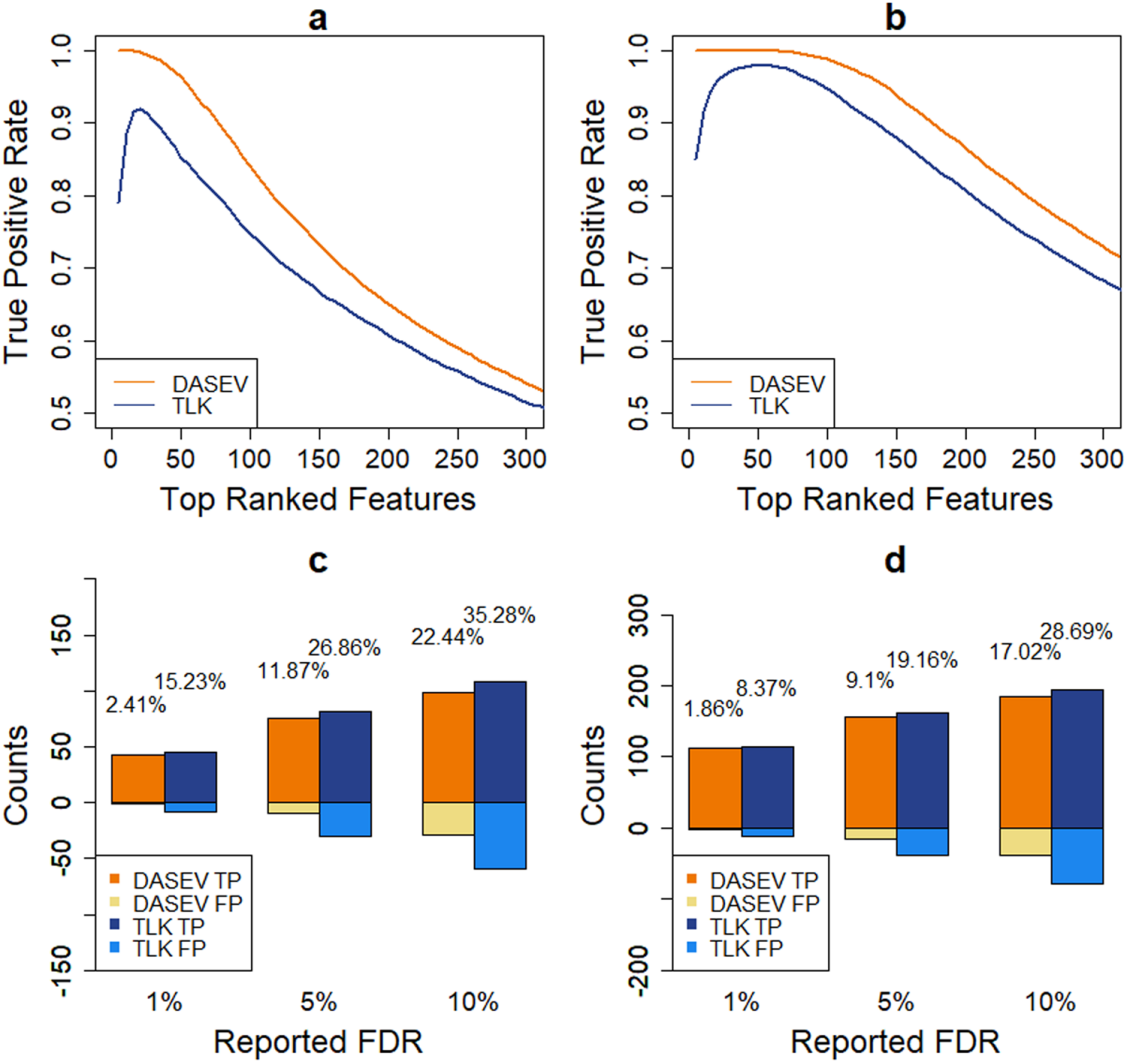
Comparison of differential abundance analysis results from DASEV and TLK based on simulations from the first scenario. Panels a and b are the true positive rate of top-ranked features with a sample size of 100 and 200 per group, respectively. Panels c and d are numbers of true positive (TP) and false positive (FP) features for a reported FDR threshold of 1%, 5% or 10% with a sample size of 100 and 200 per group, respectively. The percentage shown on top of a bar is the observed FDR. The results were averaged from 100 simulations.

We next compared the performance of DASEV and TLK for identifying differentially abundant features comparing the two groups, where we focused on testing ${H}_{0}^{M}$. One important task in this endeavor is to rank features based on their likelihood of being differentially abundant. Figure 1 shows the top 150 features ranked by each method based on one simulated dataset. Within the top features identified by TLK, 23 (15%) were false positives. The corresponding true positive rate (TPR) was 85%. Most of those features had large fractions of PMVs. Their estimated variances tended to be very small, some were even close to zero (Fig. 1a). Therefore, TLK assigned favorable rankings to them even though some of them had very small fold changes. In contrast, none of the top-ranked features from DASEV had very small estimated variance (Fig. 1b). There were only 12 (8%) false positives. The corresponding TPR was 92%. Note that although most of the features with very small estimated variances by TLK were false positives, there were two true positive features. Those two features had large fractions of PMVs, and their true variances were much larger than the estimated values. TLK gave very small variance estimates to many features with large fractions of PMVs, which happened to include those two true positive features. Since TLK tended to rank features with very small estimated variances to the top, those two true positive features were identified along with other false positive features. It should be pointed out that this particular simulated dataset we chose is representative. The TPR of the top 150 features for each method based on this dataset was close to the average TPR over 100 simulated datasets (Fig. 4b). In addition, we checked several other simulated datasets. The difference between DASEV and TLK was also observed (results not shown). To get a more general understanding for the improvement of DASEV over TLK, we plotted the TPR as a function of the number of selected top-ranked features. The resulting curve based on an average over 100 simulations is presented in Fig. 4. For DASEV, the TPR was one for the very top-ranked features and decreased as more features were included. In contrast, the TPR curve based on TLK was not monotonic, indicating that even the very top features selected by TLK contained false positives. The TPR curve based on DASEV was higher than that based on TLK, indicating DASEV was able to rank more true positive features to the top compared to TLK. As we increased the sample size from 100 (Fig. 4a) to 200 (Fig. 4b) per group, the TPR also increased for both methods, with the TPR based on DASEV still higher than that based on TLK.

In addition, we compared numbers of true positive and false positive features identified by DASEV and TLK for a given FDR threshold, referred to as the reported FDR, of 1%, 5%, or 10%. Figure 4c,d present the results averaged over 100 simulations for sample size of 100 and 200 per group, respectively. Compared to TLK, DASEV identified similar numbers of true positives but much fewer false positives. For example, for simulations with 100 observations per group and at 1% FDR threshold, DASEV identified an average of 43 true positives and only 1 false positives. In contrast, TLK identified an average of 45 true positives and 8 false positives. The numbers of identified features increased as sample size increased to 200 per group for both DASEV and TLK. But DASEV still had much fewer false positives. For both methods, the observed FDR, which was calculated as the observed fraction of false positives among identified features, was higher than the reported FDR. But the observed FDR from DASEV was much closer to the reported FDR than that from TLK. In addition, the observed FDR became closer to the reported FDR for both methods as the sample size increased from 100 to 200 per group.

We also performed the test for ${H}_{0}^{B}$, and compared DASEV to two additional parametric methods: accelerated failure time model (AFT)^5^ and two-part t-test (2T)^3,15^. DASEV still had the best performance under such a situation (Supplementary Fig. S8). In addition to the first simulation scenario presented here, we considered five other simulation scenarios, see Supplementary Table S1 for a summary of those scenarios. The second scenario aimed to evaluate DASEV’s performance in testing difference in BPMV proportions. We considered hypothesis testing for both ${H}_{0}^{P}$ and ${H}_{0}^{B}$. DASEV outperformed TLK in testing ${H}_{0}^{P}$ (Supplementary Fig. S5). For testing ${H}_{0}^{B}$, AFT yielded the highest TPR curve (Supplementary Fig. S9), but it was likely due to mis-characterizing the difference in zero proportions between groups as a difference in means of lognormal distributions, see Additional Simulation Results section in Supplementary Materials for details. Our third to fifth scenarios considered the situation where both BPMV proportion and non-BPMV mean abundance are different between groups. In reality, differentially abundant features can be either dissonant (lower BPMV proportion with lower mean for non-BPMVs) or consonant (higher BPMV proportion with lower mean for non-BPMVs)^3^. Therefore, we considered the following three scenarios: equal amount of dissonant and consonant features (the third scenario), more dissonant features (the fourth scenario), and more consonant features (the fifth scenario). Our third to fifth scenarios aimed to assess the situations where both BPMV proportions and group means are different using different proportion of dissonant and consonant features to represent those situations respectively. DASEV outperformed other methods in most situations (Supplementary Fig. S10 to S12). The only exception was that for the fifth scenario with small sample size (10 or 20 per group), AFT performed slightly better than DASEV. This again was likely due to mis-characterizing the difference in BPMV proportions as a difference in non-BPMV means, see Additional Simulation Results section in Supplementary Materials for details. Our sixth simulation scenario was designed to examine methods’ performance while no TPMVs were present. As shown in Supplementary Fig. S13, DASEV performed the best among all methods. More detailed descriptions of simulation settings and results are provided in Supplementary Materials.

### Human urinary proteome data analysis

**Figure 5:**
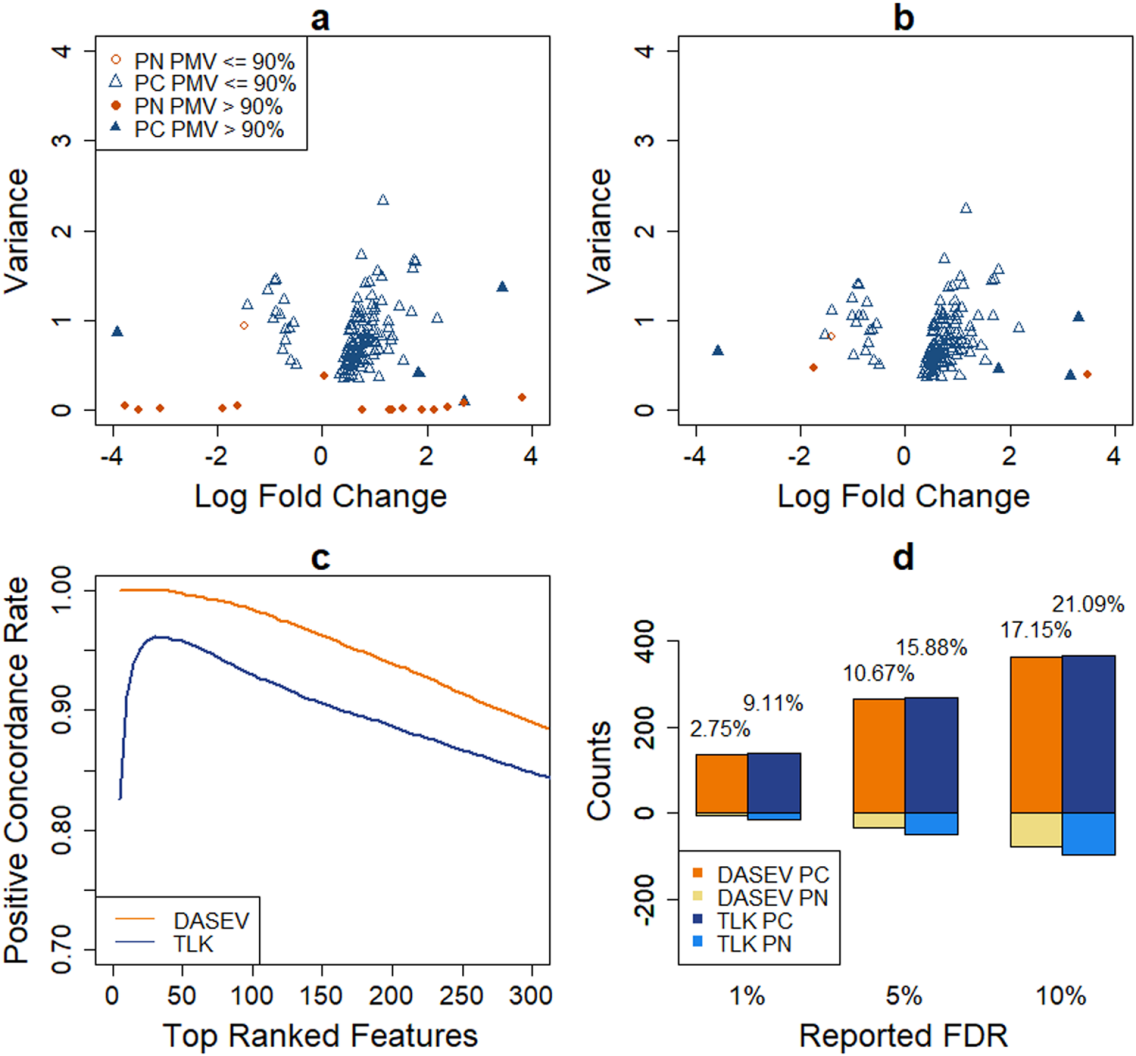
Comparison of DASEV and TLK based on subsampling 100 observations per group from the human urinary proteome dataset. Panel a and b are estimated log fold change versus variance for TLK and DASEV, respectively. Panel c shows the positive concordance rate between the subsample and full dataset. Panel d shows numbers of positive concordance (PC) and positive non-concordance (PN) features based on the subsample analysis for a reported FDR threshold of 1%, 5% or 10%. The percentage shown on top of a bar is the observed positive non-concordance rate. For panels a and b, results were based on a single subsample. For panels c and d, results were averaged across 100 subsamples.

The human urinary proteome database (HUPD) contains proteomic data of urine samples analyzed by capillary electrophoresis-mass spectrometry from 13,027 patients with 47 different pathophysiological conditions^6^. For demonstrating our method, we focused on a subset of 362 benign prostatic hyperplasia patients and 1,503 healthy controls with the goal of identifying differentially abundant peptide features between these two groups. In our analysis, we considered a total of 5,270 features that had at least three non-PMV observations and at least one PMV and one non-PMV observation per group. The range of PMV proportion for this dataset was from 0.7% to 99.8% with 80.8% as the mean value. The first and third quantiles were 75.6% and 95.5%, respectively. For demonstration, we focused on testing the hypothesis ${H}_{0}^{M}$ and applied both DASEV and TLK. Under the threshold of FDR ≤ 0.01, DASEV identified 971 and TLK identified 1017 significant features, within which 963 were identified by both methods. We next performed a subsample analysis to investigate the concordance between full data analysis and subsample analysis. Specifically, we considered the 963 features commonly identified by both methods from the full dataset as the set of “positive” features, denoted by ${\mathscr{P}}$. Likewise, we obtained 2726 features with FDR q-value > 0.3 and considered them as the set of “negative” features, denoted by ${\mathscr{N}}$. We focused only on those “positive” and “negative” features and randomly sampled 100 or 200 observations from each of the benign prostatic hyperplasia and healthy control groups to form a subsample. We applied DASEV and TLK to the subsample to assess whether they could recover the “positive” and “negative” features obtained from the full dataset. Results from subsampling 100 observations per group were shown in Fig. 5. Those from subsampling 200 observations per group were provided in Supplementary Fig. S6. Among the 150 top-ranked features, DASEV identified 11 more features in ${\mathscr{P}}$ than TLK (Fig. 5a,b. Similar to our findings from simulation studies, the variance estimates based on TLK were very close to zero for a number of features, which explained the higher number of TLK identified features that were not in ${\mathscr{P}}$. In addition, the positive concordance rate, defined as the fraction of identified features that were in ${\mathscr{P}}$, based on DASEV was always about 10% higher than that based on TLK at any given threshold of the number of top-ranked features (Fig. 5c). Furthermore, at a given FDR threshold, DASEV identified similar numbers of features in ${\mathscr{P}}$ yet fewer features in ${\mathscr{N}}$ compared to TLK (Fig. 5d). All these analyses suggested that the subsample analysis results from DASEV had a higher concordance with the full data analysis results.

### Non-small cell lung cancer exosomal lipids data analysis

**Figure 6:**
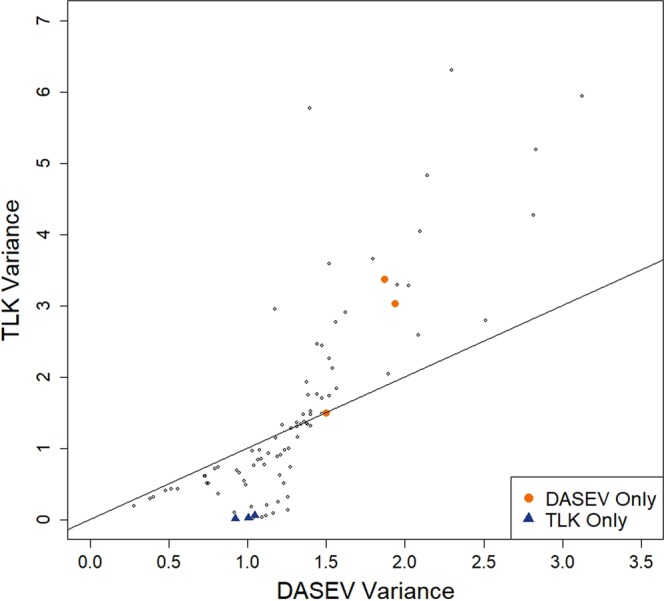
Comparison of Non-small cell lung cancer exosomal lipids data analysis results between DASEV and TLK. Estimated variances from these two methods are plotted against each other for the 101 lipid features. The solid line indicates where the two estimates are equal. Orange dots indicate the three differentially abundant features only identified by DASEV and blue triangles indicate the three differentially abundant features only identified by TLK.

For a second independent real sample type, we analyzed the lipid profile dataset of exosomes isolated from non-small cell lung cancer patient plasma reported by Fan *et al*.^7^. We focused on identifying differentially abundant lipids features between 44 early stage and 47 late stage lung cancer patients. Our analysis considered 101 features that had at least three non-PMV observations and at least one PMV and one non-PMV observation per group. The molecular formulae of the compounds were determined by ultra high-resolution Fourier transform MS as previously described^7^. We applied both DASEV and TLK to the dataset and focused on testing the hypothesis ${H}_{0}^{M}$. Each method returned 11 significant features at reported FDR < 10%. Among them, 8 features were identified by both methods. Figure 6 compares variance estimates between TLK and DASEV. The range of estimated variances based on DASEV was much narrower than that based on TLK. There were no extremely large or small variance estimates based on DASEV. We specifically investigated the variance estimates for the features detected only by one method. Blue triangles are for the 3 features detected only by TLK. The TLK estimated variances were close to zero for all these three features, which was the reason that TLK called them as significant. In contrast, the DASEV estimated variances were larger, and therefore these features were not top-ranked by DASEV. Orange dots are for the 3 features detected only by DASEV. For two of these features, TLK returned large estimated variances, and thus failed to identify them. For the remaining feature, C52H76O6, TLK and DASEV gave similar variance estimates. This feature ranked #12 in TLK with an FDR q-value of 0.1278, which barely missed the FDR threshold. One reason was that TLK ranked the three features with underestimated variances ahead of C52H76O6, and therefore moved C52H76O6 above the FDR threshold.

Using the program PREMISE^16^, these features were assigned to lipidic components as shown in Table 1. Most of the lipids are triglycerides and glycerophospholipids. Triglycerides are typically storage lipids. However, some of the acyl chains of the TAGs are polyunsaturated, which can be hydrolyzed to bioactive lipids (diacylglycerols and the fatty acid). Studies have shown that fatty acid biosynthetic pathways can be molecular targets for cancer therapy, including lung cancer^17^. Glycerophospholipids and their lyso forms are seen to be differentially abundant in the plasma of the cancer patients^18^. One compound (C35H69N2O6P1), which was identified only by DASEV, belongs to sphingolipids. Sphingolipids are implicated in important cell signaling processes and are known to have regulatory roles in tumor growth, including lung cancer^19,20^.

**Table 1:** Lipid information for compounds identified in differential abundance analysis by DASEV and TLK.

Formula	Lipid group	Lipid class	Acyl chain	Unsat sites	# carbons	DASEV p-value	TLK p-value	DASEV q-value	TLK q-value	DASEV variance	TLK variance	PMV %
C56H102O6*	Triacylglycerols	TAG	53	3	56	0.0005	0.0004	0.0486	0.0103	0.9783	0.5492	91.2
C18H34N1O9P1*	Lysoglycerophospholipids	LysoPS	12	1	18	0.0044	0.0047	0.0896	0.0475	2.5112	2.7940	63.7
C30H58N1O6P1*	Ceramides	Cer-1P	30	2	30	0.0038	0.0004	0.0896	0.0103	1.0351	0.0206	96.7
C42H84N1O8P*	Glycerophospholipids	PC	34	0	42	0.0029	0.0029	0.0896	0.0324	1.2781	1.2790	79.1
C44H84N1O8P1*	Glycerophospholipids	PC	36	2	44	0.0020	0.0015	0.0896	0.0306	0.3807	0.2972	8.8
C44H82N1O8P1*	Glycerophospholipids	PC	36	3	44	0.0081	0.0064	0.0972	0.0586	0.4035	0.3138	22.0
C44H88N1O8P1*	Glycerophospholipids	PC	36	0	44	0.0077	0.0004	0.0972	0.0103	0.9188	0.0977	95.6
C66H106O6*	Triacylglycerols	TAG	63	11	66	0.0096	0.0023	0.0972	0.0324	1.0275	0.1820	96.7
C35H69N2O6P1^*D*^	Sphingolipids	SM	30	2	35	0.0092	0.0232	0.0972	0.1617	1.8705	3.3688	95.6
C52H76O6^*D*^	Triacylglycerols	TAG	49	12	52	0.0066	0.0164	0.0972	0.1278	1.4831	1.4947	96.7
C55H82O6^*D*^	Triacylglycerols	TAG	52	12	55	0.0106	0.0240	0.0976	0.1617	1.9373	3.0262	94.5
C61H104O6^*L*^	Triacylglycerols	TAG	58	7	61	0.0635	<0.0001	0.3308	0.0013	0.9219	0.0140	94.5
C45H82N1O8P1^*L*^	Glycerophospholipids	PE	40	4	45	0.0884	0.0018	0.3308	0.0311	1.0041	0.0243	94.5
C61H112O6^*L*^	Triacylglycerols	TAG	58	3	61	0.0589	0.0026	0.3304	0.0324	1.0452	0.0599	96.7

*Features identified by both DASEV and TLK. ^*D*^Features identified only by DASEV. ^*L*^Features identified only by TLK.

## Discussions

In this paper, we introduced an empirical Bayes shrinkage method to stabilize the variance estimation of mixture model for MS data in presence of a large fraction of PMVs. The stabilization of variance estimation also led to more precise estimations of the BPMV proportion and the non-BPMV mean, which are important parameters characterizing a feature’s abundance level in a group of subjects. As for differential abundance analysis to compare these parameters between groups, comparing to TLK, our method substantially increased the power of the analysis with a higher true positive rate among top-ranked features.

The empirical Bayes shrinkage method stabilizes the variance estimation by borrowing information from the ensemble of features, which is achieved by assuming a common prior distribution of variance across features. Due to the introduction of this prior, the variance estimation becomes slightly biased. As shown in Fig. 1b, DASEV underestimated the variances. However, in finite samples, especially when sample size is small, unbiasedness may not be that important. To stably estimate variance is more important, which can substantially improve the power of differential abundance analysis, see Fig. 4. In this paper, we consider an inverse chi-square distribution as the prior, which appears to fit the data (Supplementary Fig. S1). The general applicability of the inverse chi-square distribution needs to be further investigated with the accumulation of proteomics and metabolomics datasets. However, we examined the robustness of our method against the choice of the prior parametric distribution. Supplementary Fig. S7 compares the results from using lognormal versus inverse chi-square as the prior distribution. The results from the two prior distributions were similar, especially when sample size was larger. Therefore, DASEV appeared to be insensitive to the choice of prior distribution.

Regarding the variance estimation, the TLK method used a lower bound of 0.0025, which was set somewhat arbitrarily. In our simulations, we found that this lower bound was reached by some features, who tended to be identified as significant features because this lower bound value was very small. In DASEV, rather than specifying a lower bound, we introduced a prior distribution for the variances across features. The prior distribution does not specify a lower bound so that any positive number is theoretically allowed. However, the parameters in the prior distribution are adaptively (empirically) selected to favor variance values that are reasonable for the data by assigning higher prior probabilities to those values, and thus substantially reduces the chance of getting extremely large or small variance estimates. Since the prior distribution is empirically determined from the data, it is sufficiently flexible to fit various datasets of different biological variations such as human data, mice data, and cell line data.

In practice, we recommend to focus on features having at least three non-PMVs for two group comparison because under this restriction, at least one group can have 2 or more non-PMV observations so that there is information about the variance of the feature abundance. However, one can choose alternative restrictions. On one side, DASEV can still be applied to features with less than three non-PMVs. Under such situations, the data may not contain variation information of the feature. But our shrinkage method allows borrowing information from other features to estimate the variance, although this is not ideal. On the other side, one may focus on features with a decent number of non-PMVs, which contain richer information on the distribution of non-PMVs. Based on the first simulation scenario, we compared three different restrictions that requires at least two, three, or ten non-PMVs. Results are provided in Supplementary Fig. S14 (for sample size of 20 per group) and S15 (for sample size of 100). The overall performance was similar between the restrictions of at least two and at least three non-PMVs for each of the DASEV, TLK, AFT, and 2T methods. When applying the more stringent restriction of at least 10 non-PMVs, the performance (in terms of TPR) of DASEV, TLK and 2T all improved, while that of AFT remained about the same. Notably, under such situations, performances of DASEV, TLK and 2T were very close to each other. This is as expected because when there are a decent number of non-PMVs, the variance estimation becomes stable, and does not depend much on the choice of the estimation method.

In our algorithm, we have noticed two convergence issues. The first issue was sometimes observed when using the R function *optim* to find maximum likelihood estimates or during the iterative procedure for model parameter estimation. We were able to solve this issue by increasing the maximum number of iterations.The second issue was sometimes observed for estimating BPMV proportions and fitting the model under a perfect separation, i.e. a group only has PMVs while the other group only has non-PMVs. This was solved in our two-group comparison analysis by restricting features to have at least one PMV and one non-PMV in each group. The issue can appear at a higher frequency when categorical covariates are included in the model. We therefore recommend to check on the perfect separation issue before applying the method.

We consider a lognormal model for non-BPMVs and a logistic model for BPMVs in this paper, which is most commonly used in MS data analysis. Our method can be extended to other models, such as a probit model for the BPMV proportion and a skew normal model for non-BPMVs^21^. Under these models, we expect the empirical Bayes shrinkage method can still be used to improve model parameter estimation.

The large amount of PMVs in an MS dataset causes challenges in not only differential abundance analysis but also other types of statistical analysis, such as cluster analysis. Traditional methods for cluster analysis, e.g. the k-mean and hierarchical clustering methods, do not specifically handle zero values. The mixture model provides a promising alternative. Extending our method to cluster analysis is one of our future research interests.

## Supplementary information

Supplementary information .

## Data Availability

The non-small cell lung cancer exosomal lipids dataset is available at the NIH Common Fund’s National Metabolomics Data Repository (NMDR) website, the Metabolomics Workbench, https://www.metabolomicsworkbench.org, where it has been assigned Project ID PR000854. The data can be accessed directly via it’s Project DOI: 10.21228/M8998T.

## Code availability

An R package, *dasev*, and simulation analysis code used in this paper are available at http://sweb.uky.edu/cwa236/DASEV.html. The R code for TLK and AFT was downloaded from the publication website https://www.degruyter.com/view/j/sagmb.2013.12.issue-6/sagmb-2013-0021/Appendix2_corrected.pdf. The R code for 2T was from Taylor and Pollard^15^. All analyses were done in RStudio Version 1.2.1335 with R 3.5.3.
