# Supplementary material for: Differential Abundance Analysis with Bayes Shrinkage Estimation of Variance (DASEV) for Zero-Inflated Proteomic and Metabolomic Data
Source: Sci Rep. 2020 Jan 21;10:876. doi: 10.1038/s41598-020-57470-4 (PMC6972855; doi:10.1038/s41598-020-57470-4)
Supplement: Supplementary file 1 — Supplementary information . [file 41598_2020_57470_MOESM1_ESM.pdf]

Supplementary Materials for "Differential Abundance Analysis with Bayes Shrinkage Estimation of Variance (DASEV) for Zero-Inflated Proteomic and Metabolomic Data"

Zhengyan Huang[1], Andrew N. Lane[2,3,4], Teresa W-M. Fan[2,3,4], Richard M. Higashi[2,3,4], Heidi L. Weiss[1,2], Xiangrong Yin[5], Chi Wang[1,2,*]

[1] Department of Biostatistics, University of Kentucky, Lexington, Kentucky, 40536, USA
[2] Markey Cancer Center, University of Kentucky, Lexington, Kentucky, 40536, USA
[3]Center for Environmental and Systems Biochemistry, University of Kentucky, Lexington, Kentucky, 40536, USA
[4]Department of Toxicology and Cancer Biology, University of Kentucky, Lexington, Kentucky, 40536, USA
[5]Department of Statistics, University of Kentucky, Lexington, Kentucky, 40536, USA
[*]Corresponding author E-mail address: chi.wang@uky.edu(Chi Wang)

Calculation of Hyperparameters

We assume σ_k^2 follows an inverse-gamma distribution with shape parameter $d_0/2$ and scale parameter $d_0 s_0^2/2$.

$$\sigma_k^2 \sim \Big(\frac{d_0 s_0^2}{2}\Big)^{\frac{d_0}{2}} \frac{\sigma_k^{2\left(-1-\frac{d_0}{2}\right)}}{\Gamma(\frac{d_0}{2})} \exp\Big(-\frac{d_0 s_0^2}{2\sigma_k^2}\Big).$$

The mean and variance of the inverse-gamma distribution are

$$E(\sigma_k^2) = \frac{d_0 s_0^2/2}{d_0/2-1}.$$

$$Var(\sigma_k^2) = E((\sigma_k^2)^2) - (E(\sigma_k^2))^2 = \frac{(d_0 s_0^2/2)^2}{(d_0/2-1)^2(d_0/2-2)}.$$

Let m and v be the sample mean and smaple variance, respectively. Based on the method of moments, s_0 and d_0 can be calculated as:

$$d_0 = \frac{2m^2}{v} + 4.$$

$$s_0 = \sqrt{\frac{m(d_0-2)}{d_0}}.$$

Scenario	LFC	OR	TPMV	Ratio	Results
1	log(2)	1	Random	—	Main text Figure 1 to 4. Supplementary Figures S2, S3 S4, S7, S8, S14, and S15.
2	0	2	Random	—	Supplementary Figures S5, S9, and S16.
3	log(2)	2	Random	1:1	Supplementary Figure S10.
4	log(2)	2	Random	82:18	Supplementary Figure S11.
5	log(2)	2	Random	18:82	Supplementary Figure S12.
6	log(2)	1	0	—	Supplementary Figure S13.

Table S1: A list of simulation scenarios. LFC: log fold change between control and case group mean abundance of non-BPMVs. OR: odds ratio between control and case group BPMV proportions. TPMV: TPMV proportions. TPMV proportions were obtained based on randomly selected BPMV proportions, mean abundance, detect limit, and variance. Ratio: ratio of dissonant vs consonant features.

Additional Simulation Results

We performed additional simulation studies to more comprehensively assess the performance of DASEV under various practical situations. A list of the six simulation scenarios considered in this paper is provided in Table S1.

Under the first scenario, other than testing for H_0^M that is presented in the main text, we also performed test for H_0^B. We considered both smaller ($n=$ 10 or 20 per group) and larger ($n=$ 100 or 200 per group) sample sizes, and compared DASEV to two additional parametric methods, accelerated failure time model (AFT)[1] and two part t-test (2T)[2, 3]. The AFT method assumes all PMVs are TPMVs and characterizes the data by a log-normal distribution with a detection limit. The 2T method integrates a chi-square test to compare PMV proportion and a t-test to compare log-transformed non-PMVs. DASEV had better performance than AFT and 2T under both larger and smaller sample size situations (Supplementary Figure S8). DASEV held the highest TPR among the very top-ranked features. As the number of top-ranked features increased, 2T obtained similar performance. AFT's performance did not improve much by increasing the sample size. All other methods performed much better with larger sample size. Although AFT held lower observed FDR for sample size 100 and 200 per group, the counts of TPs were much smaller than other methods. DASEV obtained reasonable counts of TPs and observed FDR at all three thresholds.

The second scenario aimed to evaluate DASEV's performance in testing difference in BPMV proportions. We considered hypothesis testing for both H_0^P and H_0^B. DASEV outperformed TLK in testing H_0^P (Supplementary Figure S5). DASEV was able to identify more truly differentially abundant features than TLK. The observed FDR from DASEV was smaller than the threshold while the observed FDR from TLK was much larger than the threshold for the

sample size of 100. As the sample size increased to 200, the observed FDR for both methods became closer to the thresholds. For testing H_0^B, AFT yielded the highest TPR curve (Supplementary Figure S9). AFT also resulted the best observed FDR and counts of TPs. However, when we examined the estimation of log fold change among differentially abundant features, the estimation from AFT had a much larger deviation from the true value compared to the other methods (Supplementary Figure S16). In contrast, among features which were not differentially abundant, all four methods showed similar deviation. Therefore, the difference in zero proportion between groups were mis-characterized as difference in the mean of lognormal distribution by AFT since it considered all zero values as left censored from a lognormal distribution. Thus, although AFT appeared to have high performance in identifying differentially abundant features, the inference from AFT was unreliable under such situations. Compare to AFT, DASEV obtained better estimation on group means. In addition, DASEV performed better than TLK and 2T.

Our third to fifth scenarios considered the situation where both BPMV proportions and non-BPMV mean abundance are different between groups. In reality, differentially abundant features can be either dissonant (lower BPMV proportion with lower mean for non-BPMVs) or consonant (higher BPMV proportion with lower mean for non-BPMVs). Therefore, we considered the following three scenarios: equal amount of dissonant and consonant features (the third scenario), more dissonant features (the fourth scenario), and more consonant features (the fifth scenario). For each scenario, 10% features were randomly selected as differentially abundant features. In the third scenario, we set a ratio of 1:1 for differentially abundant features to have higher versus lower mean abundance in the case group than in the control group. We also set a ratio of 1:1 for those features to have higher versus lower BPMV proportion in case group compared to the control group. In the fourth scenario, we set both ratios to 9:1. In the fifth scenario, we set the ratio for differentially abundant features to have higher versus lower mean abundance in the case group to 9:1, and the ratio for those features to have higher versus lower BPMV proportion in case group to 1:9. As a result, the ratios between dissonant and consonant features were 1:1 , 82:18, and 18:82 for those three scenarios, respectively. Simulation results for testing H_0^B are presented in Supplementary Figure S10 to S12. All methods performed better when there were more consonant features (Supplementary Figures S11 and S12). TPR curves were generally lower if the data contained more dissonant features. The observed FDRs were similar for scenarios with more dissonant or more consonant features. However, counts of TPs were much higher in the later scenario for sample size 100 and 200 per group. DASEV outperformed other methods in most situations (Supplementary Figure S10 to S12). The only exception was that for the fifth scenario with small sample size of 10 or 20 per group (Supplementary Figure S12), AFT performed slightly better than DASEV. This again is likely due to mis-characterizing difference in BPMV proportions as difference in non-BPMV means, which happened to increase the difference in non-BPMV means for consonant features. Therefore, for a dataset dominated by consonant features as we showed in the fifth scenario, AFT had

higher power. However, for dissonant features, the difference in non-BPMV means caused by mis-characterization of difference in BPMV proportions were in the opposite direction to the true difference in non-BPMV means so that those two would cancel out. As shown by the fourth scenario (Supplementary Figure S11), when there were more dissonant features, AFT had the lowest performance compared to other methods. We also explored numbers of consonant and dissonant features identify by each method when equal amount of those features existed (the third scenario). AFT identified more consonant features and the other three methods resulted similar amount of consonant and dissonant features (results not shown).

Our sixth simulation scenario was designed to examine methods' performance while no TPMVs were present. DASEV outperformed all other methods (Supplementary Figure S13). The results show a similar pattern to what we obtained for the first simulation scenario (Supplementary Figure S8). DASEV had the highest TPR among the very top-ranked features. As the number of top-ranked features increased, 2T obtained similar performance as DASEV. All methods other than AFT performed much better with larger sample size. Although AFT held lower observed FDR for sample size 100 and 200 per group, The counts of TPs were much smaller than other methods. DASEV obtained reasonable number of TPs and observed FDR at all three FDR thresholds.

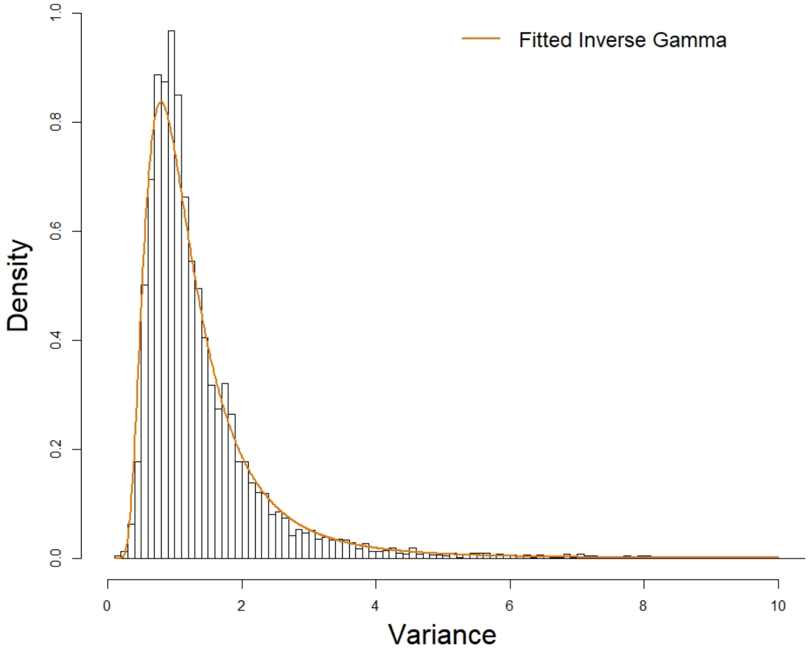

Figure S1: Empirical distribution of $\hat{\sigma}_k^2$ from the human urinary proteome dataset. The orange fitted curve was from an inverse-gamma distribution $\mathit{Inv\text{-}Gamma}(d_0/2; d_0 s_0^2/2)$, where d_0 and s_0 were calculated as described in the main text of the paper.

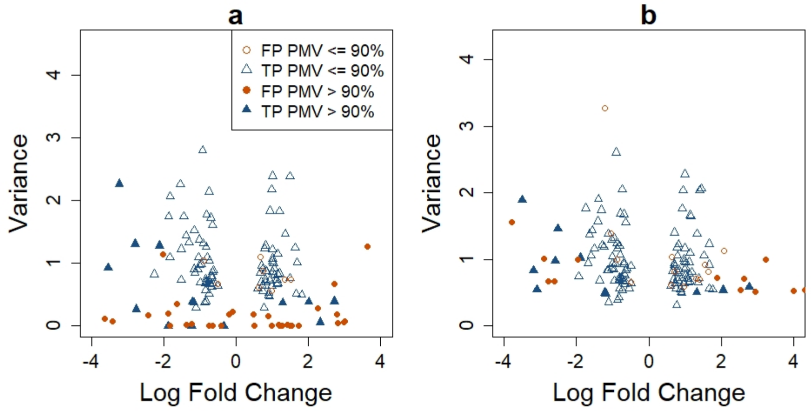

Figure S2: Estimated log fold change versus variance based on TLK (panel a) and DASEV (panel b) for 150 top-ranked features from a two-group differential abundance analysis. Data were simulated as described in the Simulation Studies section with a sample size of 100 per group from the first scenario. Features were ranked based on their p-values. FP: false positive; TP: true positive.

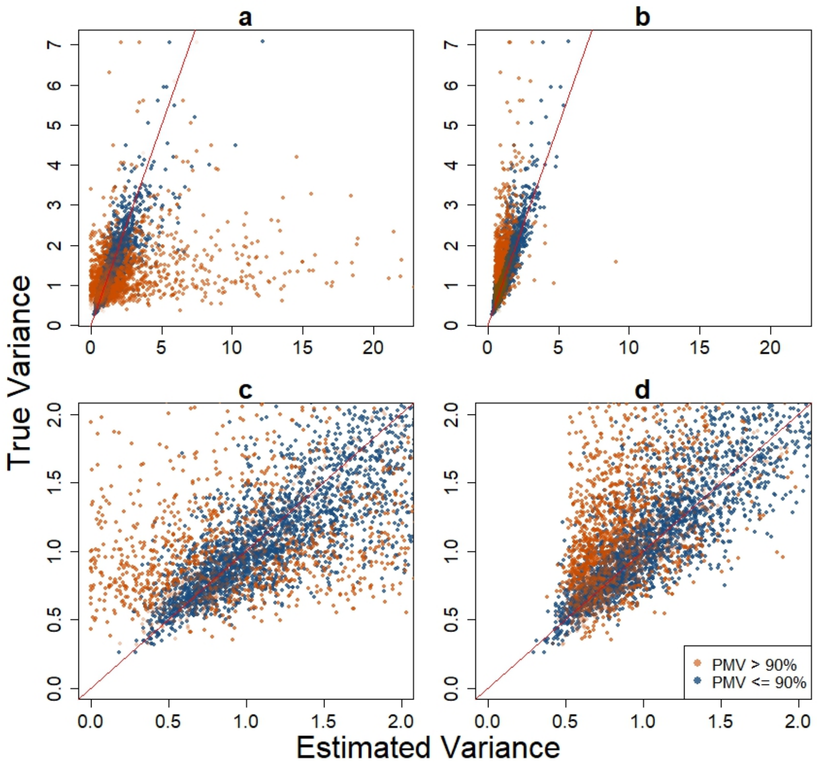

Figure S3: Comparison of estimated variance versus true variance for TLK (panels a and c) and DASEV (panels b and d) based on a single simulation with sample size 100 per group from the first scenario. Panels c and d are magnified lower left corner of panels a and b, respectively. The red line shows where the estimated variance equals the true variance.

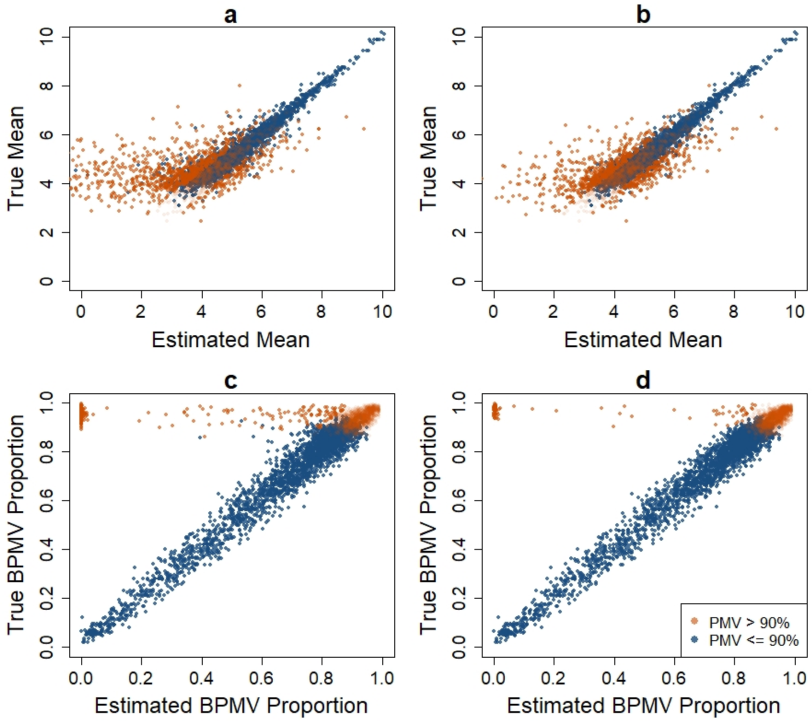

Figure S4: Comparison of estimated non-BPMV mean and BPMV proportion versus true values for TLK (panels a and c) and DASEV (panels b and d) based on a single simulation with a sample size of 100 per group from the first scenario. This figure only shows results for the control group. The case group had identical patterns.

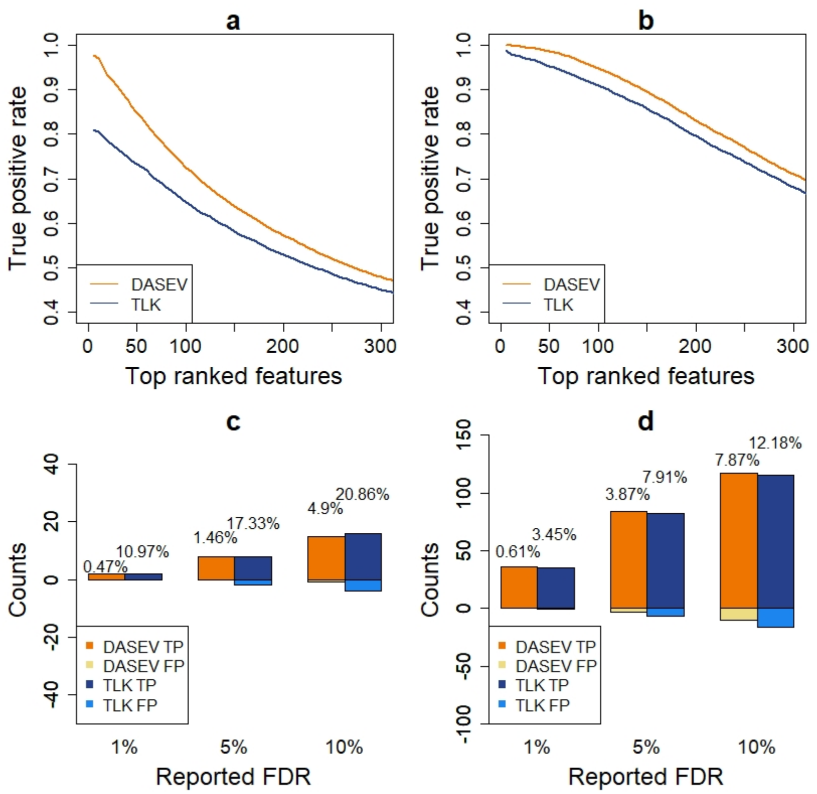

Figure S5: Comparison of differential abundance analysis results from DASEV and TLK for testing H_0^P. Data were generated based on the second simulation scenario. Panels a and b are the true positive rate of top-ranked features with a sample size of 100 and 200 per group, respectively. Panels c and d are numbers of true positive (TP) and false positive (FP) features for a reported FDR threshold of 1%, 5% or 10% with a sample size of 100 and 200 per group, respectively. The percentage shown on top of a bar is the observed FDR. The results were averaged from 100 simulations.

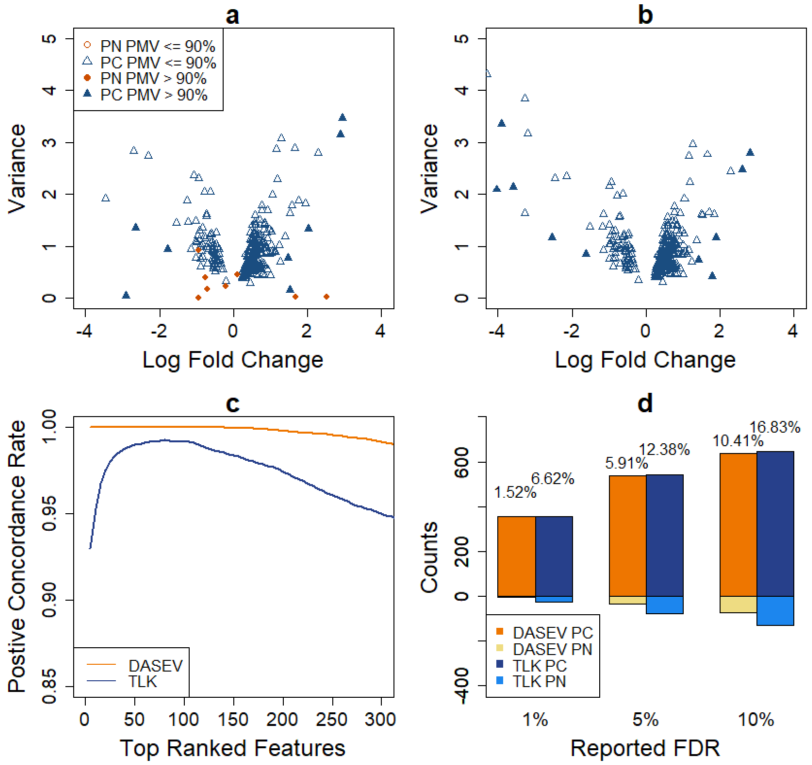

Figure S6: Comparison of DASEV and TLK on testing H_0^M based on subsampling 200 observations per group from the human urinary proteome dataset. Panel a and b are estimated log fold change versus variance for TLK and DASEV, respectively. Panel c shows the positive concordance rate between the subsample and full dataset. Panel d shows numbers of positive concordance (PC) and positive non-concordance (PN) features based on the subsample analysis for a reported FDR threshold of 1%, 5% or 10%. The percentage shown on top of a bar is the true positive non-concordance rate. For panels a and b, results were based on a single subsample. For panels c and d, results were averaged across 100 subsamples.

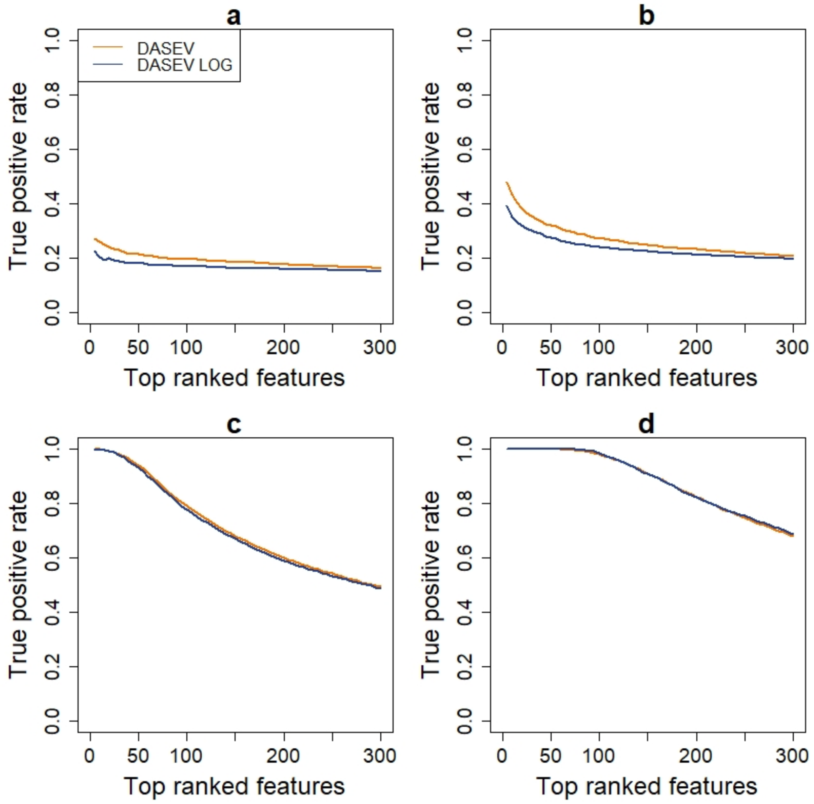

Figure S7: Comparison of DASEV with inverse gamma (DASEV) and log normal (DASEV LOG) as prior distribution for variance. Panels a to d are the true positive rate of top-ranked features with a sample size of 10, 20, 100 and 200 per group, respectively. The results were averaged from 100 simulations based on the first simulation scenario.

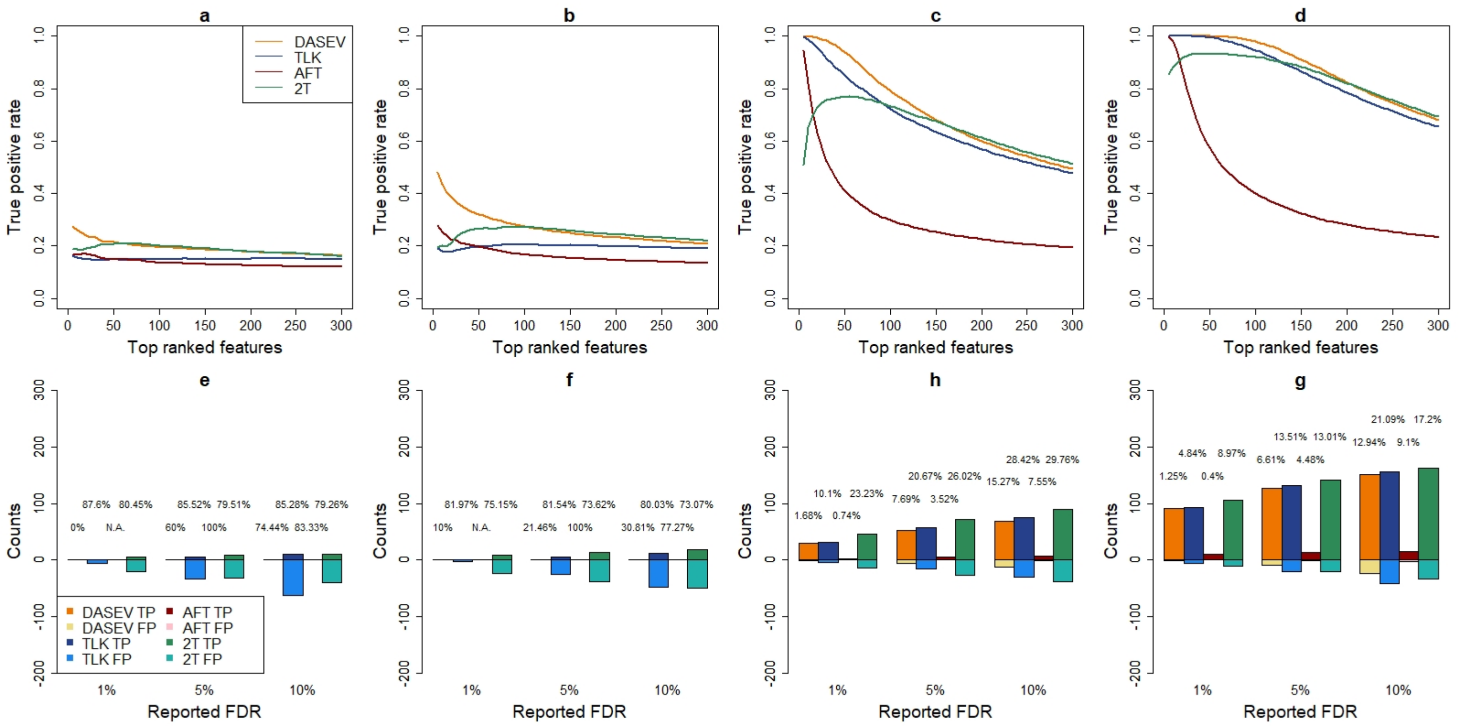

Figure S8: Comparison of differential abundance analysis results from DASEV, TLK, AFT, and 2T. Results for DASEV, TLK, and 2T were obtained for testing H_0^B. Data were generated based on the first simulation scenario. Panels a to d are the true positive rate of top-ranked features with a sample size of 10, 20, 100 and 200 per group, respectively. Panels e to h are numbers of true positive (TP) and false positive (FP) features for a reported FDR threshold of 1%, 5% or 10% with a sample size of 10, 20, 100 and 200 per group, respectively. The percentage shown on top of a bar is the observed FDR. FDR was calculated only using simulations with at least one feature identified at each threshold. N.A. means no features were identified over all 100 simulations. The results were averaged from 100 simulations.

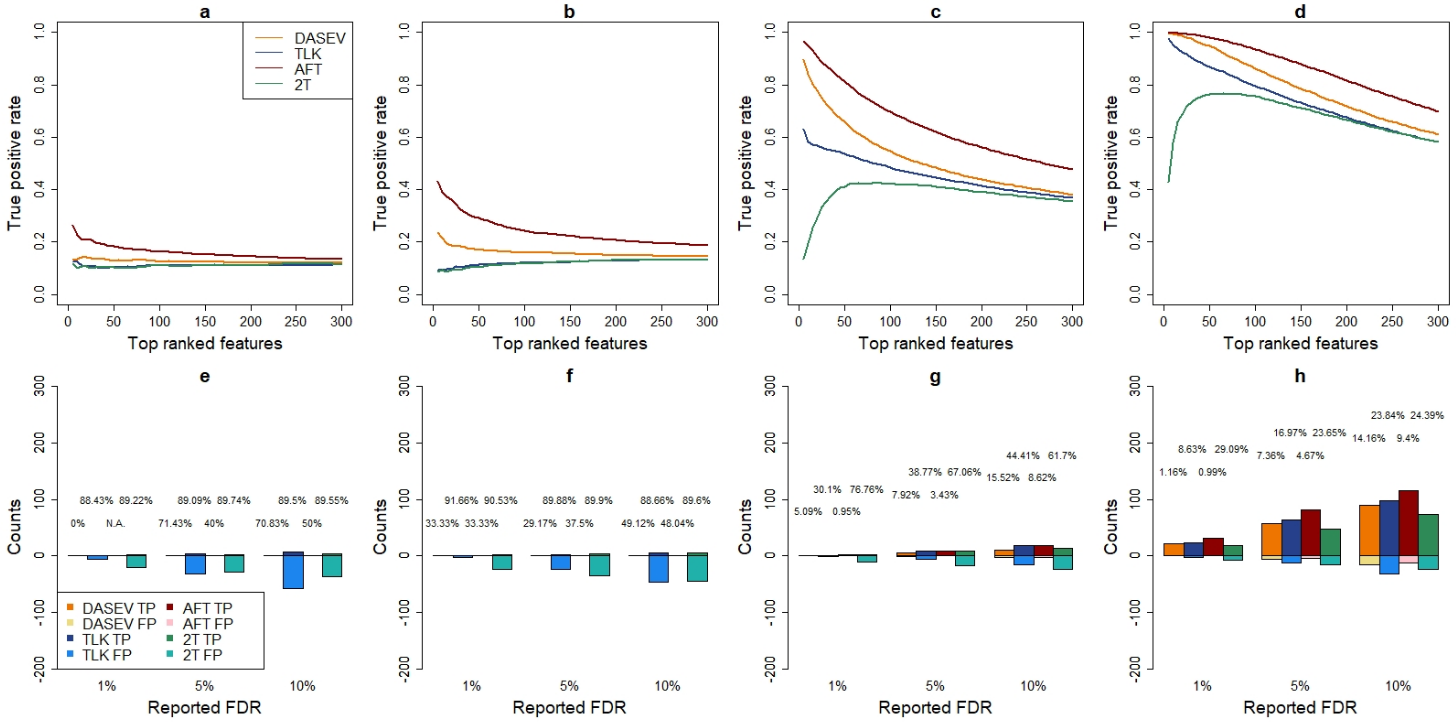

Figure S9: Comparison of differential abundance analysis results from DASEV, TLK, AFT, and 2T. Results for DASEV, TLK, and 2T were obtained for testing H_0^B. Data were generated based on the second simulation scenario. Panels a to d are the true positive rate of top-ranked features with a sample size of 10, 20, 100 and 200 per group, respectively. Panels e to h are numbers of true positive (TP) and false positive (FP) features for a reported FDR threshold of 1%, 5% or 10% with a sample size of 10, 20, 100 and 200 per group, respectively. The percentage shown on top of a bar is the observed FDR. FDR was calculated only using simulations with at least one feature identified at each threshold. N.A. means no features were identified over all 100 simulations. The results were averaged from 100 simulations.

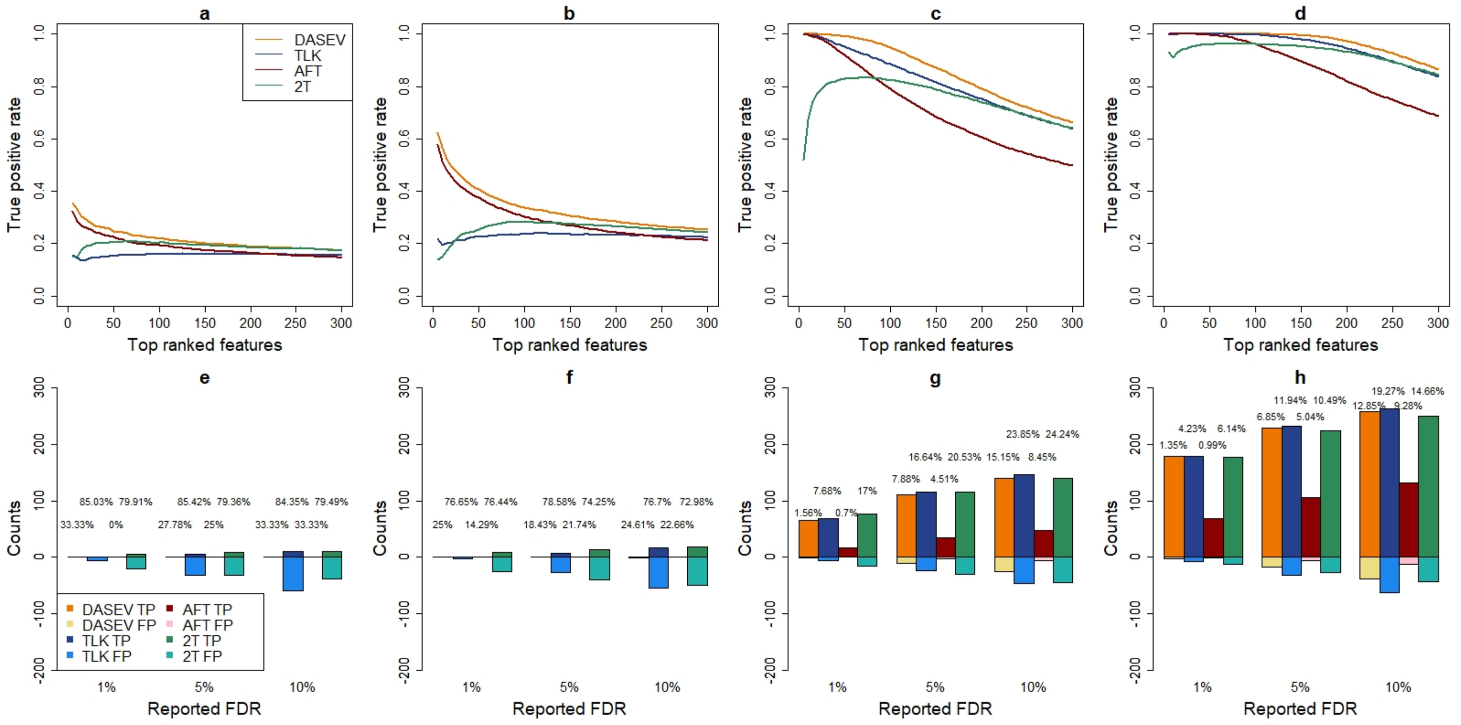

Figure S10: Comparison of differential abundance analysis results from DASEV, TLK, AFT, and 2T. Results for DASEV, TLK, and 2T were obtained for testing H_0^B. Data were generated based on the third simulation scenario. Panels a to d are the true positive rate of top-ranked features with a sample size of 10, 20, 100 and 200 per group, respectively. Panels e to h are numbers of true positive (TP) and false positive (FP) features for a reported FDR threshold of 1%, 5% or 10% with a sample size of 10, 20, 100 and 200 per group, respectively. The percentage shown on top of a bar is the observed FDR. FDR was calculated only using simulations with at least one feature identified at each threshold. The results were averaged from 100 simulations.

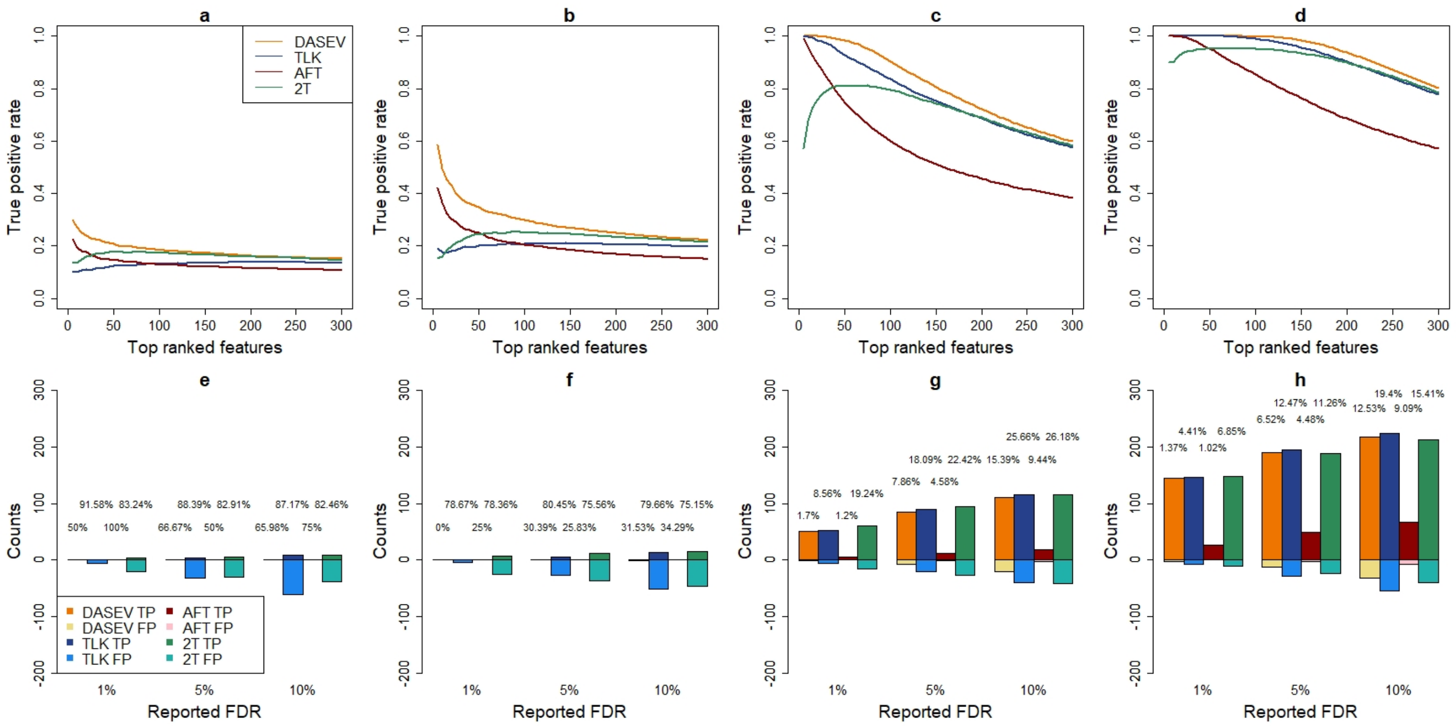

Figure S11: Comparison of differential abundance analysis results from DASEV, TLK, AFT, and 2T. Results for DASEV, TLK, and 2T were obtained for testing H_0^B. Data were generated based on the fourth simulation scenario with more dissonant features. Panels a to d are the true positive rate of top-ranked features with a sample size of 10, 20, 100 and 200 per group, respectively. Panels e to h are numbers of true positive (TP) and false positive (FP) features for a reported FDR threshold of 1%, 5% or 10% with a sample size of 10, 20, 100 and 200 per group, respectively. The percentage shown on top of a bar is the observed FDR. FDR was calculated only using simulations with at least one feature identified at each threshold. The results were averaged from 100 simulations.

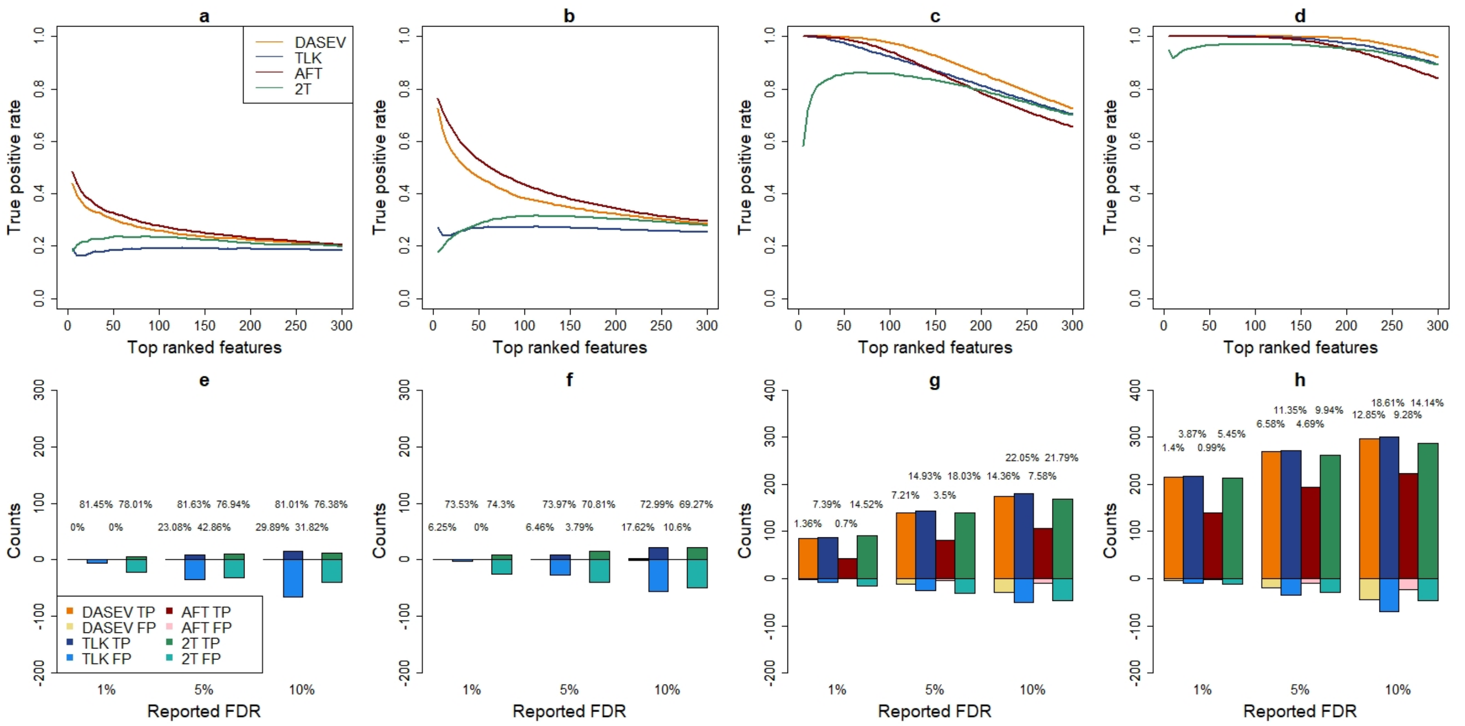

Figure S12: Comparison of differential abundance analysis results from DASEV, TLK, AFT, and 2T. Results for DASEV, TLK, and 2T were obtained for testing H_0^B. Data were generated based on the fifth simulation scenario with more consonant features. Panels a to d are the true positive rate of top-ranked features with a sample size of 10, 20, 100 and 200 per group, respectively. Panels e to h are numbers of true positive (TP) and false positive (FP) features for a reported FDR threshold of 1%, 5% or 10% with a sample size of 10, 20, 100 and 200 per group, respectively. The percentage shown on top of a bar is the observed FDR. FDR was calculated only using simulations with at least one feature identified at each threshold. The results were averaged from 100 simulations.

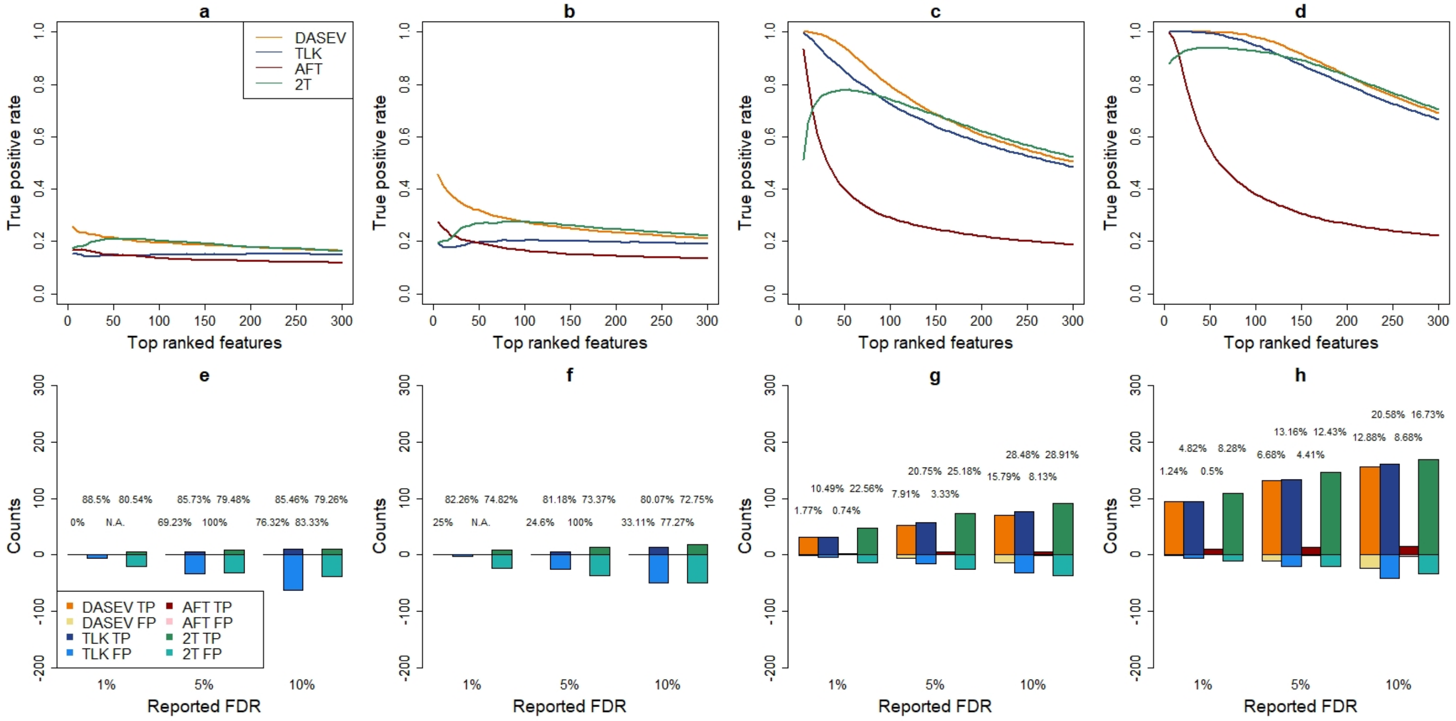

Figure S13: Comparison of differential abundance analysis results from DASEV, TLK, AFT, and 2T. Results for DASEV, TLK, and 2T were obtained for testing H_0^B. Data were generated based on the sixth simulation scenario. Panels a to d are the true positive rate of top-ranked features with a sample size of 10, 20, 100 and 200 per group, respectively. Panels e to h are numbers of true positive (TP) and false positive (FP) features for a reported FDR threshold of 1%, 5% or 10% with a sample size of 10, 20, 100 and 200 per group, respectively. The percentage shown on top of a bar is the observed FDR. FDR was calculated only using simulations with at least one feature identified at each threshold. N.A. means no features were identified over all 100 simulations. The results were averaged from 100 simulations.

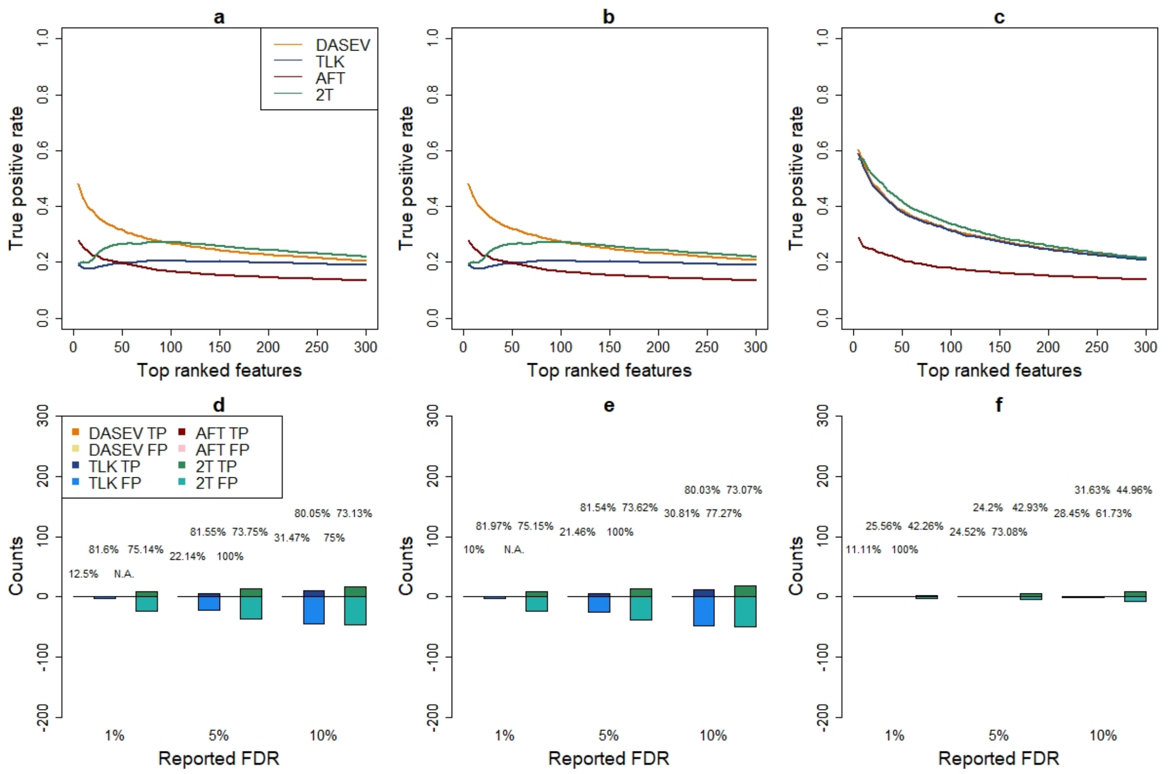

Figure S14: Comparison of differential abundance analysis results from DASEV, TLK, AFT, and 2T. Results for DASEV, TLK, and 2T were obtained for testing H_0^B. Data were generated based on the first simulation scenario with sample size 20 per group. Panels a to c are the true positive rate of top-ranked features for simulations with at least 2, 3, and 10 non-PMV observations, respectively. Panels d to f are numbers of true positive (TP) and false positive (FP) features for a reported FDR threshold of 1%, 5% or 10% for simulations with at least 2, 3, and 10 non-PMV observations, respectively. The percentage shown on top of a bar is the observed FDR. FDR was calculated only using simulations with at least one feature identified at each threshold. N.A. means no features were identified over all 100 simulations. The results were averaged from 100 simulations.

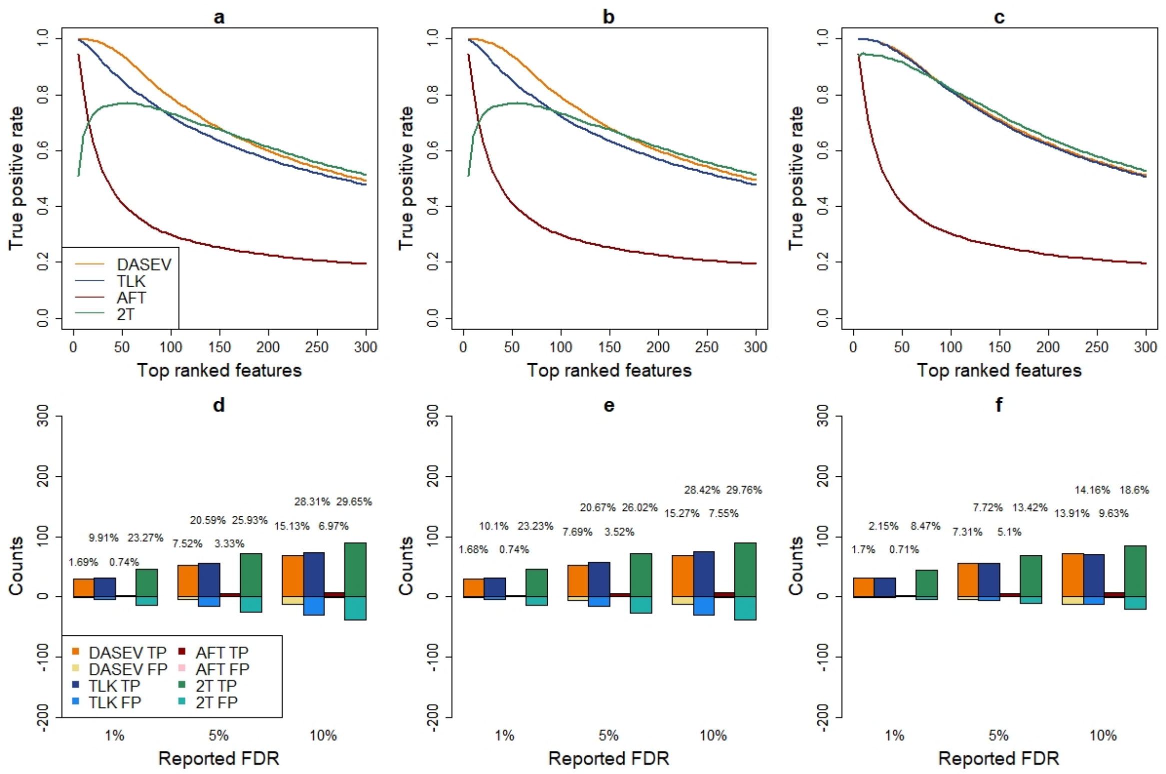

Figure S15: Comparison of differential abundance analysis results from DASEV, TLK, AFT, and 2T. Results for DASEV, TLK, and 2T were obtained for testing H_0^B. Data were generated based on the first simulation scenario with sample size 100 per group. Panels a to c are the true positive rate of top-ranked features for simulations with at least 2, 3, and 10 non-PMV observations, respectively. Panels d to f are numbers of true positive (TP) and false positive (FP) features for a reported FDR threshold of 1%, 5% or 10% for simulations with at least 2, 3, and 10 non-PMV observations, respectively. The percentage shown on top of a bar is the observed FDR. FDR was calculated only using simulations with at least one feature identified at each threshold. The results were averaged from 100 simulations.

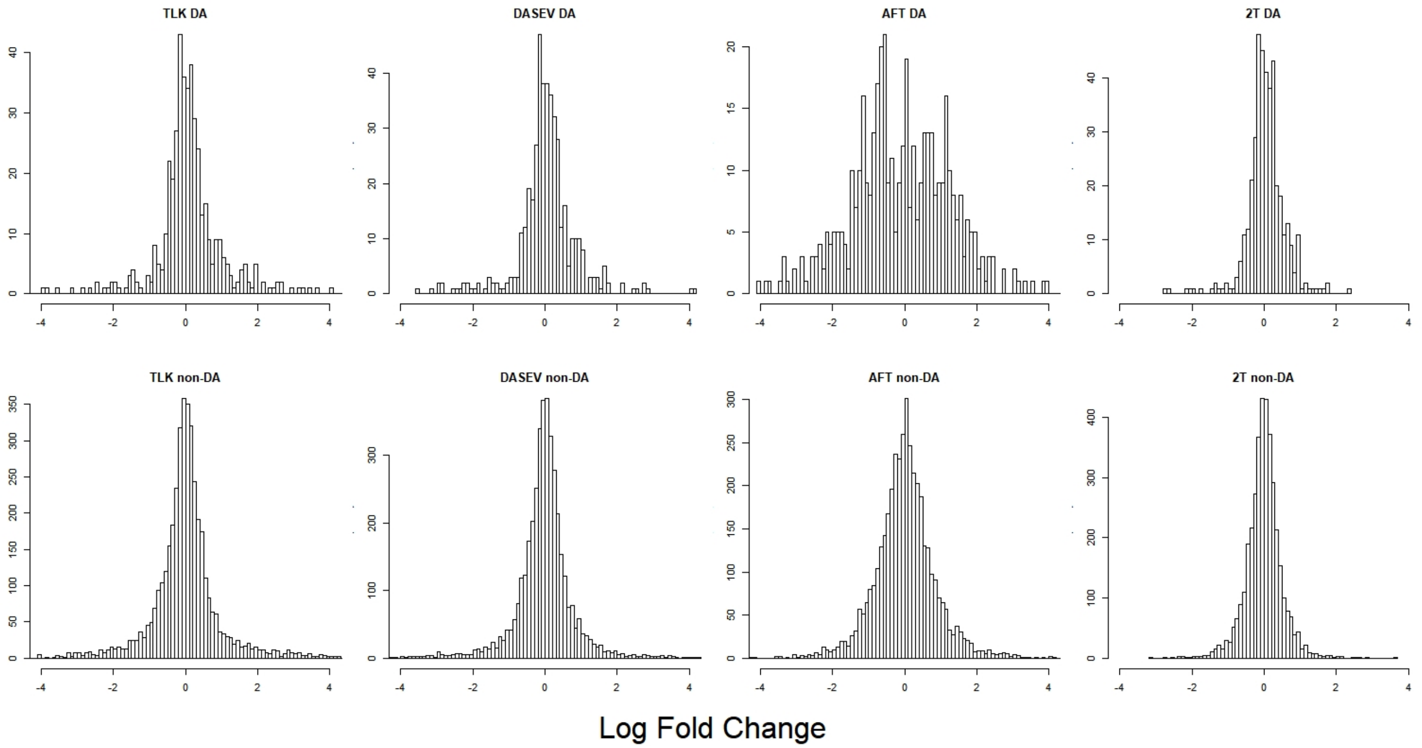

Figure S16: Comparison of Log Fold Change estimates obtained from DASEV, TLK, AFT, and 2T. Data were generated based on the second simulation scenario with sample size of 100 per group. Panels a to d are the distribution of LFC for true differential abundant (DA) features. Panels e to h are the distribution of LFC for true non-differential abundant (non-DA) features. Panel c suggests that AFT had larger variance in mean difference for differential abundant features, which should only differ in BPMV proportions. The other three methods obtained similar distribution for mean difference among differential abundant and non-differential abundant features.

References

[1] Sandra L Taylor, Gary S Leiserowitz, and Kyoungmi Kim. Accounting for undetected compounds in statistical analyses of mass spectrometry 'omic studies. *Statistical applications in genetics and molecular biology*, 12(6):703—722, December 2013.

[2] Sandra L Taylor, , and K. Pollard. Hypothesis tests for point-mass mixture data with application to 'omics data with many zero values. *Statistical applications in genetics and molecular biology*, 8, 2009.

[3] Andreas Gleiss, Mohammed Dakna, Harald Mischak, and Georg Heinze. Two-group comparisons of zero-inflated intensity values: the choice of test statistic matters. *Bioinformatics*, 31:2310–2317, 2015.
